# Deletion of MCP-1 Impedes Pathogenesis of Acid Ceramidase Deficiency

**DOI:** 10.1038/s41598-018-20052-6

**Published:** 2018-01-29

**Authors:** Fabian P. S. Yu, Shaalee Dworski, Jeffrey A. Medin

**Affiliations:** 1 Institute of Medical Science, University of Toronto, Toronto, ON Canada; 2 Department of Medical Biophysics, University of Toronto, Toronto, ON Canada; 3 University Health Network, Toronto, ON Canada; 4 Department of Pediatrics, Medical College of Wisconsin, Milwaukee, WI USA; 5 Department of Biochemistry, Medical College of Wisconsin, Milwaukee, WI USA

## Abstract

Farber Disease (FD) is an ultra-rare Lysosomal Storage Disorder caused by deficient acid ceramidase (ACDase) activity. Patients with ACDase deficiency manifest a spectrum of symptoms including formation of nodules, painful joints, and a hoarse voice. Classic FD patients will develop histiocytes in organs and die in childhood. Monocyte chemotactic protein (MCP-1; CCL2) is significantly elevated in both FD patients and a mouse model we previously generated. Here, to further study MCP-1 in FD, we created an ACDase;MCP-1 double mutant mouse. We show that deletion of MCP-1 reduced leukocytosis, delayed weight loss, and improved lifespan. Reduced inflammation and fibrosis were observed in livers from double mutant animals. Bronchial alveolar lavage fluid analyses revealed a reduction in cellular infiltrates and protein accumulation. Furthermore, reduced sphingolipid accumulation was observed in the lung and liver but not in the brain. The neurological and hematopoietic defects observed in FD mice were maintained. A compensatory cytokine response was found in the double mutants, however, that may contribute to continued signs of inflammation and injury. Taken together, targeting a reduction of MCP-1 opens the door to a better understanding of the mechanistic consequences of ceramide accumulation and may even delay the progression of FD in some organ systems.

## Introduction

The gene *ASAH1* encodes acid ceramidase (ACDase, EC 3.5.1.23), a hydrolase that degrades the bioactive lipid ceramide into sphingosine and a free fatty acid^1^. Mutations in *ASAH1* can result in acid ceramidase deficiency which may manifest as Farber Disease (FD, Farber Lipogranulomatosis, OMIM #22000), a rare inherited Lysosomal Storage Disorder (LSD). While patients who are diagnosed with FD can display a range of symptoms, the cardinal signs are painful joints, formation of subcutaneous nodules, and the development of a hoarse voice that may lead to aphonia. In severe cases, patients may have neurologic involvement, develop hepatosplenomegaly, and fail to thrive, leading to childhood lethality^2,3^. We previously generated a mouse model of ACDase-deficiency that has high fidelity with the human disorder^4^. In this model, the P361R mouse mutation (which is orthologous to the human P362R mutation) was knocked in^5^. Mice homozygous for the mutation (*Asah1*^P361R/P361R^) recapitulate the classical form of human FD with a phenotype that includes systemic ceramide accumulation, increased leukocytosis, and enlargement of visceral organs^4^. The mice are much smaller than littermates and progressive macrophage infiltration is present in the pulmonary, hematopoietic, hepatic, and central nervous systems^4^. Large foamy macrophages and associated tissue injury is also commonly observed.

The mouse model of FD that we developed also demonstrates a unique cytokine profile that includes dramatically higher levels of MCP-1 in plasma^6^. Further, we have also recently shown that FD patients have increased levels of monocyte chenoattractant protein (MCP-1), keratinocyte chemoattractant (KC), macrophage inflammatory protein-1α (MIP-1α), and the inflammatory cytokine interferon gamma-induced protein-10 (IP-10)^6^ in their plasma. In the mouse, MCP-1 is the most elevated and its highest concentration is detected at the end of the animal’s life. MCP-1 is a potent chemoattractant that is responsible for the recruitment of monocytes. It is a member of the C-C chemokine subfamily and functions by binding to its G-protein coupled receptor: chemokine receptor 2 (CCR2). Both MCP-1 and CCR2 have been widely studied for their role in monocyte recruitment during infection and for their other roles during inflammation^7^. Expression of MCP-1 can be detected in a variety of cell types, including endothelial, smooth muscle, astrocytic, and microglial cells^8,9^. However, the major sources of MCP-1 are monocytes and macrophages^10^. Numerous FD case reports have demonstrated that histiocytic infiltration is present in many tissues and that plasma chitotriosidase is significantly elevated^3,11^. This inflammatory phenotype is reflected in our *Asah1*^P361R/P361R^ mouse, which displays macrophage and neutrophil infiltration in various tissues and organs^4,12^. In clinical practice, the management strategy for the treatment of FD focuses largely on managing patient symptoms to date. Bone marrow transplantation (BMT) may be an option for certain Farber patients as might enzyme replacement therapy (ERT)^13^. Several case reports have demonstrated that BMT reduced the number and size of subcutaneous nodules and decreased joint pain in patients with FD^14,15^. Along these lines, we previously demonstrated that MCP-1 levels are reduced in FD patient plasma post-BMT^6^. This further suggests that there may be a relationship between MCP-1 and FD symptoms, and supports MCP-1 as a prospective biomarker for FD.

Here, to further understand the role of MCP-1 in FD development and progression, we generated and characterized an *Asah1* and MCP-1/CCL2 double deficient mouse (herein called *Asah1*^P361R/P361R^;MCP-1^−/−^). In this study, we show that ablation of MCP-1 can impede symptoms of FD. This is an important insight into the pathogenesis of this disorder and opens the door to a whole new line of therapy.

## Results

### Deletion of MCP-1 improves the course of FD

**Figure 1:**
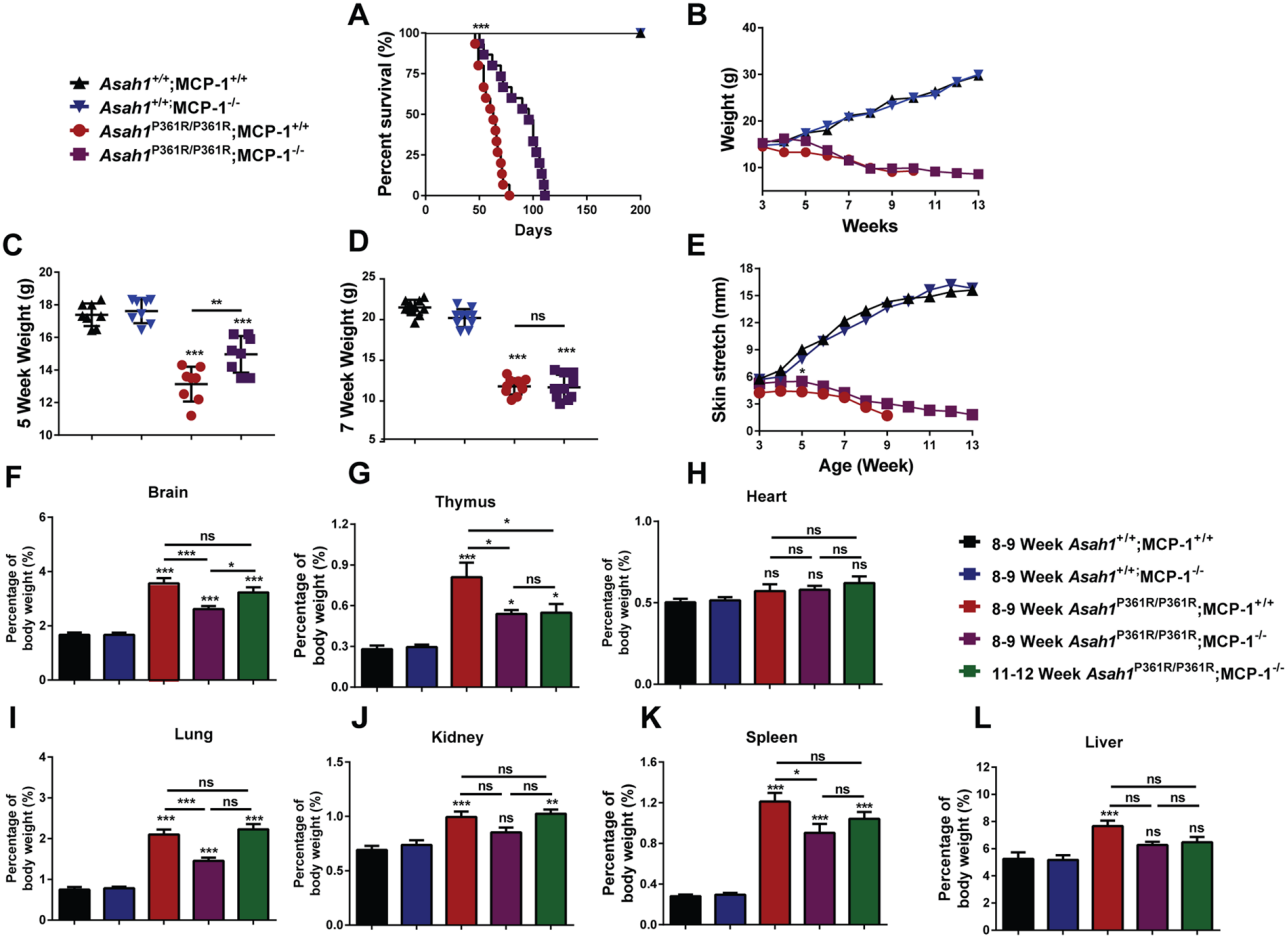
Increased survival and weight gain in *Asah1*^P361R/P361R^;MCP-1^−/−^ mice. (**A**) Kaplan-Meier survival plot (*n* = 10–15 per genotype), (**B**) Growth curve measured in weight versus age (*n* = 10 per genotype), (**C**) 5-week-old weight by genotype, (**D**) 7-week-old mice weights by genotype, (**E**) Skin stretch curves measured as length versus age (*n* = 10 per genotype). Organ weight measurements as a percentage of body weight for brain (**F**), thymus (**G**), heart (**H**), lung (**I**), kidney (**J**), spleen (**K**), and liver (**L**). *n* = 10 samples were analyzed from 8–9-weeks-old mice for each genotype and from 11–12-week-old *Asah1*^P361R/P361R^;MCP-1^−/−^ mice. All comparisons were made between 8–9-week-old mice for each genotype and samples from 11–12-week-old *Asah1*^P361R/P361R^;MCP-1^−/−^ mice. All comparisons were made between 8–9-week-old *Asah1*^+/+^;MCP-1^+/+^, ns (not significant), *p < 0.05, **p < 0.01, ***p < 0.001.

*Asah1*^P361R/P361R^;MCP-1^+/+^ mice demonstrated an expected life span of 7–9 weeks^4^. *Asah1*^P361R/P361R^;MCP-1^−/−^ mice displayed a significantly increased lifespan to a median age of 13 weeks (Fig. 1A). *Asah1*^P361R/P361R^;MCP-1^−/−^ mice also weighed more between 5 and 6 weeks of age than *Asah1*^P361R/P361R^;MCP-1^+/+^ mice (Fig. 1B,C). No differences in body weight were found after 7 weeks of age, however, between *Asah1*^P361R/P361R^;MCP-1^+/+^ and *Asah1*^P361R/P361R^;MCP-1^−/−^ mice (Fig. 1B and D). *Asah1*^P361R/P361R^; MCP-1^+/+^ mice also develop a tight skin phenotype that renders them difficult to restrain^16^. *Asah1*^P361R/P361R^;MCP-1^+/+^ and *Asah1*^P361R/P361R^;MCP-1^−/−^ mice skin stretch length was found to be significantly different at 5 weeks of age (Fig. 1E). No differences were detected in lifespan, weight, or skin stretch length between *Asah1*^P361R/P361R^; MCP-1^+/−^ and *Asah1*^P361R/P361R^;MCP-1^+/+^ mice (Supplementary Fig. S1), therefore the remainder of this study focused exclusively on homozygous *Asah1*^P361R/P361R^;MCP-1^−/−^ mice.

Organomegaly is a common phenotype present in classical FD and the *Asah1*^P361R/P361R^;MCP-1^+/+^ mouse^4^. Deletion of MCP-1 resulted in a delay in organ weight accumulation when normalized to body weight (Fig. 1). In terms of absolute organ weights, only the thymus showed a significant change in 8–9-week-old *Asah1*^P361R/P361R^;MCP-1^−/−^ mice when compared to age-matched controls (Supplementary Fig S2). The weight differences were only revealed when the organ weight was normalized with body weight. The kidney and liver weight as a percentage of total body weight in the *Asah1*^P361R/P361R^;MCP-1^−/−^ mice were within the normal ranges at 8–9-weeks of age (Fig. 1J,L). There was a decrease in brain, thymus, lung, and spleen weights as a percentage of total body weight when compared to *Asah1*^P361R/P361R^;MCP-1^+/+^ mice (Fig. 1F,G,I,K). Other than an increase in brain weight as a percentage of body weight (Fig. 1F), there were no other weight increases between 8–9 and 11–12 week-old *Asah1*^P361R/P361R^;MCP-1^−/−^ mice (Fig. 1G–L).

### Peripheral blood cell counts

**Figure 2:**
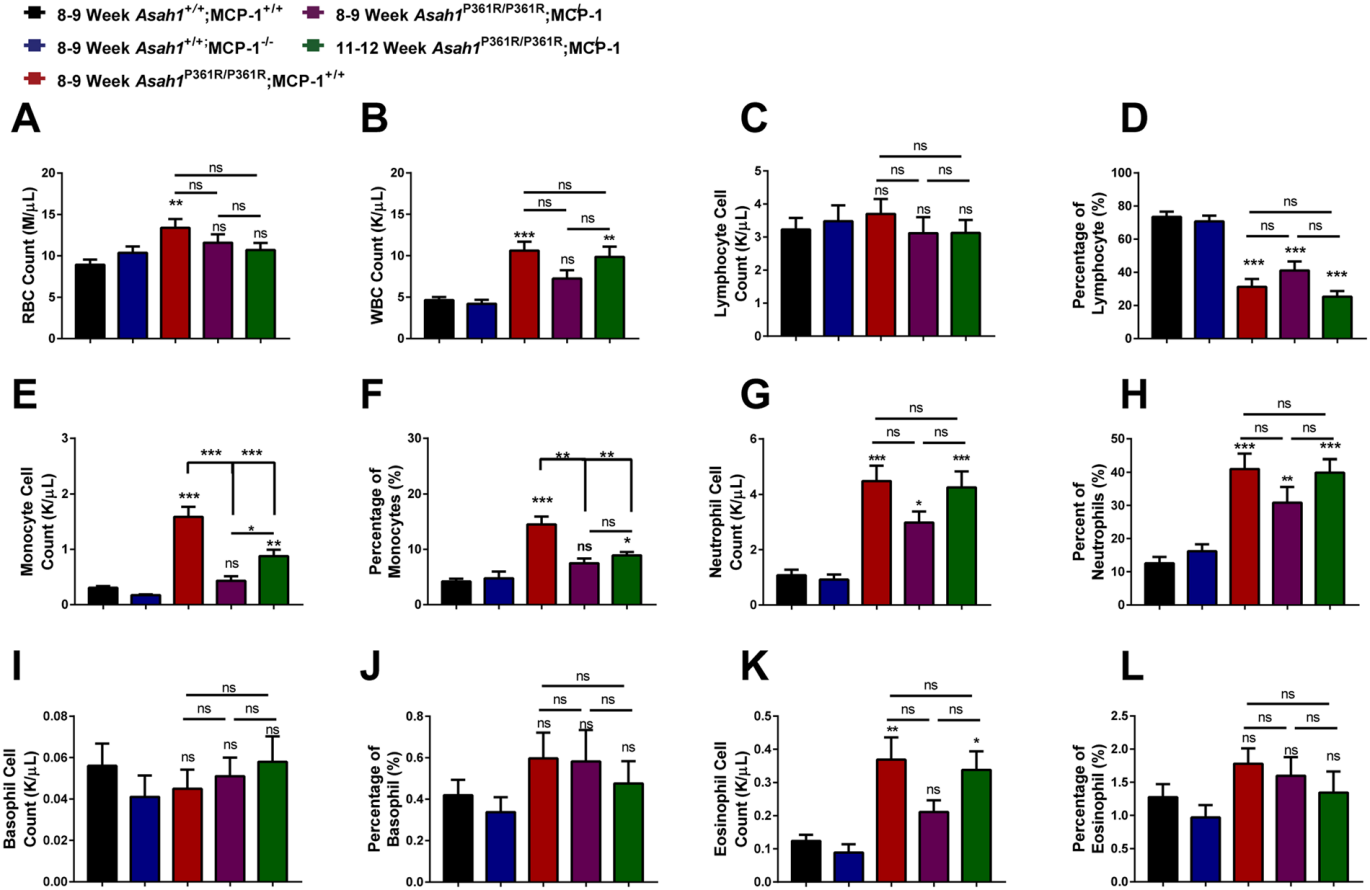
MCP-1 deletion impedes leukocytosis in ACDase-deficiency. Complete blood count (CBC) measurements showing total red blood cell (RBC) count (**A**), total white blood cell (WBC) count (**B**), lymphocyte count (**C**), lymphocyte differential (**D**), monocyte count (**E**), monocyte differential (**F**), neutrophil count (**G**), neutrophil differential (**H**), basophil count (**I**), basophil differential (**J**), eosinophil count (**K**), and eosinophil differential (**L**). *n* = 10 samples were analyzed from 8–9-week-old mice for each genotype and 11–12-week-old *Asah1*^P361R/P361R^;MCP-1^−/−^ double mutant mice. All comparisons were made between 8–9-week-old *Asah1*^+/+^;MCP-1^+/+^, *Asah1*^P361R/P361R^;MCP-1^+/+^, *Asah1*^P361R/P361R^;MCP-1^−/−^ and 11–12-week-old *Asah1*^P361R/P361R^;MCP-1^−/−^ mice. ns (not significant), *p < 0.05, **p < 0.01, ***p < 0.001.

Complete blood counts (CBCs) from peripheral blood draws of 8–9-week-old *Asah1*^P361R/P361R^;MCP-1^−/−^ mice showed a normalization in circulating red blood cells (RBC) and white blood cells (WBC) compared to 8–9-week-old *Asah1*^P361R/P361R^;MCP-1^+/+^ mice (Fig. 2A,B). However, by 11–12 weeks of age the numbers of WBCs in *Asah1*^P361R/P361R^;MCP-1^−/−^ mice were similar to that of 8–9-week-old *Asah1*^P361R/P361R^; MCP-1^+/+^ mice (Fig. 2B). Absolute lymphocyte cell counts were unchanged between genotypes; however, based on the differential cell counts of WBCs, lymphocyte percentages were significantly decreased between controls, *Asah1*^P361R/P361R^;MCP-1^+/+^, and *Asah1*^P361R/P361R^;MCP-1^−/−^ mice (Fig. 2C,D). Increased monocytes, neutrophils, and eosinophils are common in 8–9-week-old *Asah1*^P361R/P361R^;MCP-1^+/+^ mice^4^. A reduction in monocytes was found in both the absolute and differential cell counts between the 8–9-week-old *Asah1*^P361R/P361R^; MCP-1^+/+^ mice and the 8–9 and 11–12-week-old *Asah1*^P361R/P361R^;MCP-1^−/−^ mice (Fig. 2E,F). Neutrophils cell counts were not statistically significant between 8–9-week-old *Asah1*^P361R/P361R^;MCP-1^+/+^ and 8–9 and 11–12 *Asah1*^P361R/P361R^;MCP-1^+/+^ mice (Fig. 2G,H). Basophil cell counts did not change significantly either (Fig. 2I,J). Absolute eosinophil cell counts were similar to control mice in 8–9-week-old *Asah1*^P361R/P361R^;MCP-1^−/−^ mice, but were elevated in 11–12 week-old *Asah1*^P361R/P361R^;MCP-1^−/−^ mice (Fig. 2K). However, based on the differential cell counts, the percentage of eosinophils did not differ significantly between any of the genotypes (Fig. 2L).

### MCP-1 deletion does not normalize hematopoiesis

**Figure 3:**
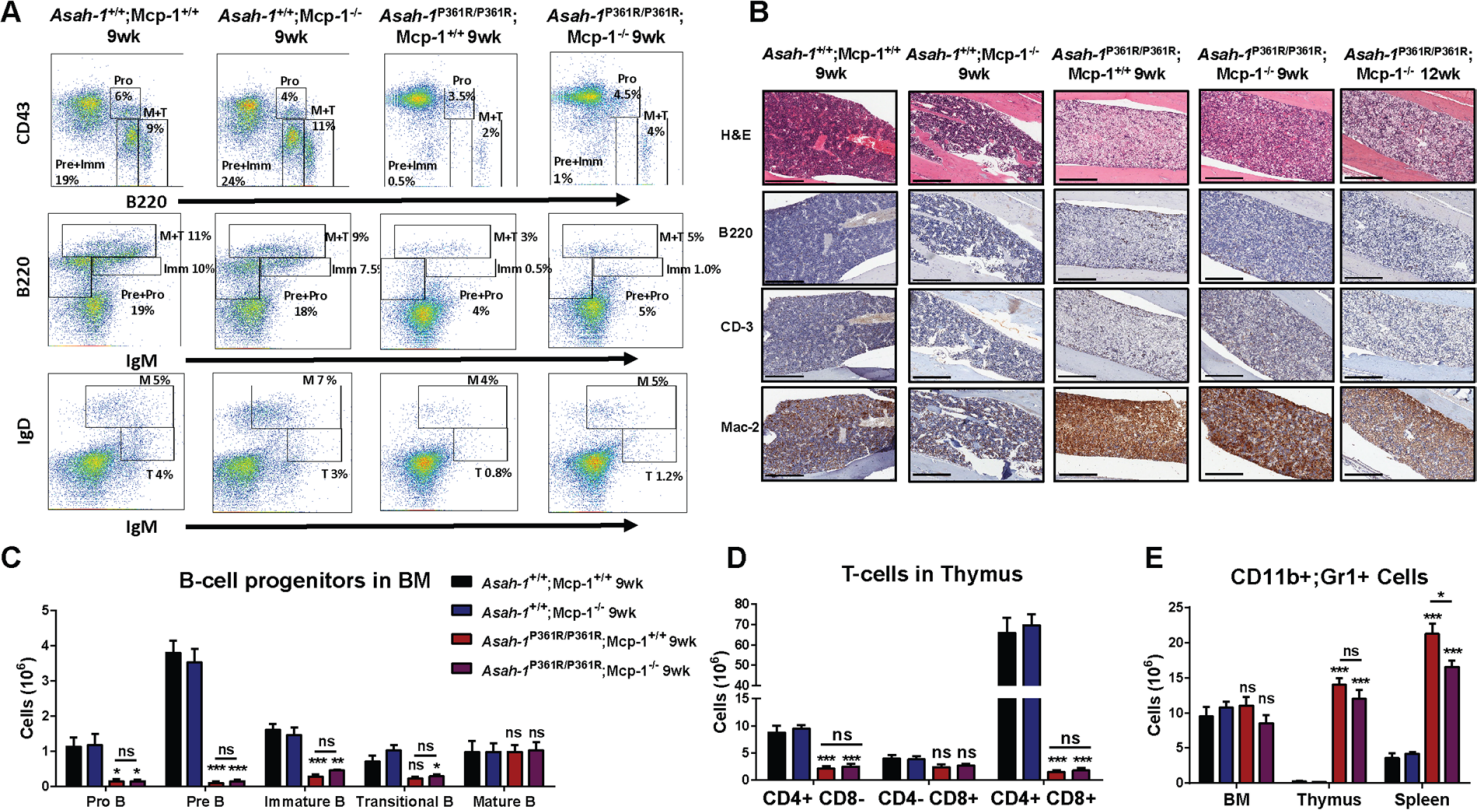
Perturbed hematopoiesis is retained in *Asah1*^P361R/P361R^;MCP-1^−/−^ mice. (**A**) FACS plots showing B-cell lineage staining in BM from 9-week-old mice of all genotypes. (**B**) Histology staining of BM with H&E along with anti-B220, anti-CD-3, and anti-Mac-2 antibodies. (**C**) The absolute number of Pro-B, Pre-B, immature B, transitional B, and mature B cells in mice bone marrow (BM). (**D**) The absolute number of CD11b^+^ and Gr-1^+^ cells in mouse BM, thymus, and spleens. (**E**) The absolute number of CD4^+^ CD8^−^, CD4^−^ CD8^+^, and CD4^+^ CD8^+^ cells in mouse thymus. 9- and 12-week-old mice were used for the histopathology analyses. Original magnification of BM micrographs at 10x where scale bar represents 300 µm. Spleen and thymus micrographs are at 4x magnification and the scale bar represents 100 µm. *n* = 3–4 mice at 9 weeks of age were used for FACS analysis and cell counts. ns (not significant), *p < 0.05 **p < 0.01, ***p < 0.001.

We previously reported that the bone marrow of *Asah1*^P361R/P361R^;MCP-1^+/+^ mice displayed a dramatic reduction in the pre-B, pro-B, immature-B, and transitional-B cells^12^. Fluorescence activated cell sorting (FACS) analysis of cells from the bone marrow of 9-week-old *Asah1*^P361R/P361R^;MCP-1^−/−^ mice also showed a reduction in B-cell progenitors (Fig. 3A). There were no differences in the various B-cell progenitor populations between 9-week-old *Asah1*^P361R/P361R^;MCP-1^+/+^ and *Asah1*^P361R/P361R^;MCP-1^−/−^ mice (Fig. 3C). H&E staining of 9-week-old *Asah1*^P361R/P361R^;MCP-1^−/−^ and both 9- and 12-week-old *Asah1*^P361R/P361R^*;MCP*-1^−/−^ tibias revealed a pale architecture compared to both *Asah*^+/+^;MCP-1^+/+^ and *Asah1*^+/+^;MCP-1^−/−^ samples. These light H&E areas were stained positive for Mac-2 but not for B220 and CD-3, which demonstrate myeloid lineage-mediated infiltration (Fig. 3B). Similarly, FACS analysis of T-cells from thymic tissue of 9-week-old *Asah1*^P361R/P361R^;MCP-1^−/−^ mice demonstrated a significant reduction in CD4^+^;CD8^+^ T-cell numbers compared to *Asah1*^P361R/P361R^;MCP-1^+/+^ mice with no differences seen between both genotypes in the various T-cell subsets (Fig. 3D and Supplementary Fig S3). Histological analysis performed on thymic tissue obtained from 9-week-old *Asah1*^P361R/P361R^;MCP-1^−/−^ mice showed pale H&E staining that was additionally characterized by the presence of Mac-2 staining. Tissue injury and macrophage infiltration appear progressive as the staining of tissue from 12 weeks of age *Asah1*^P361R/P361R^;MCP-1^−/−^ mice became more pale for H&E and more intense for Mac-2 (Supplementary Fig S3). FACS analysis of cells from 9-week-old spleen tissue of *Asah1*^P361R/P361R^; MCP-1^−/−^ mice showed a significant reduction in CD11b^+^ and Gr1^+^ granulocytes compared to cells from 9-week-old *Asah1*^P361R/P361R^;MCP-1^+/+^ mice (Fig. 3E and Supplementary Fig. S4).

The spleen parenchyma in the 9-week-old *Asah1*^P361R/P361R^;MCP-1^+/+^ mice is also highly disrupted by the presence of foamy macrophages and demonstrates a reduction in B220^+^ and CD3^+^ cells (Supplementary Fig. S4). This phenotype is present in the 9-week-old *Asah1*^P361R/P361R^;MCP-1^−/−^ mice but to a lesser extent (Supplementary Fig. S4). However, by 12 weeks of age the phenotype becomes exacerbated and mirrors that typically seen in the spleen of 9-week-old *Asah1*^P361R/P361R^;MCP-1^+/+^ mice (Supplementary Fig. S4). MCP-1 deletion also appears to decrease the rate of tissue destruction in the spleen.

### Liver inflammation and injury markers are reduced

**Figure 4:**
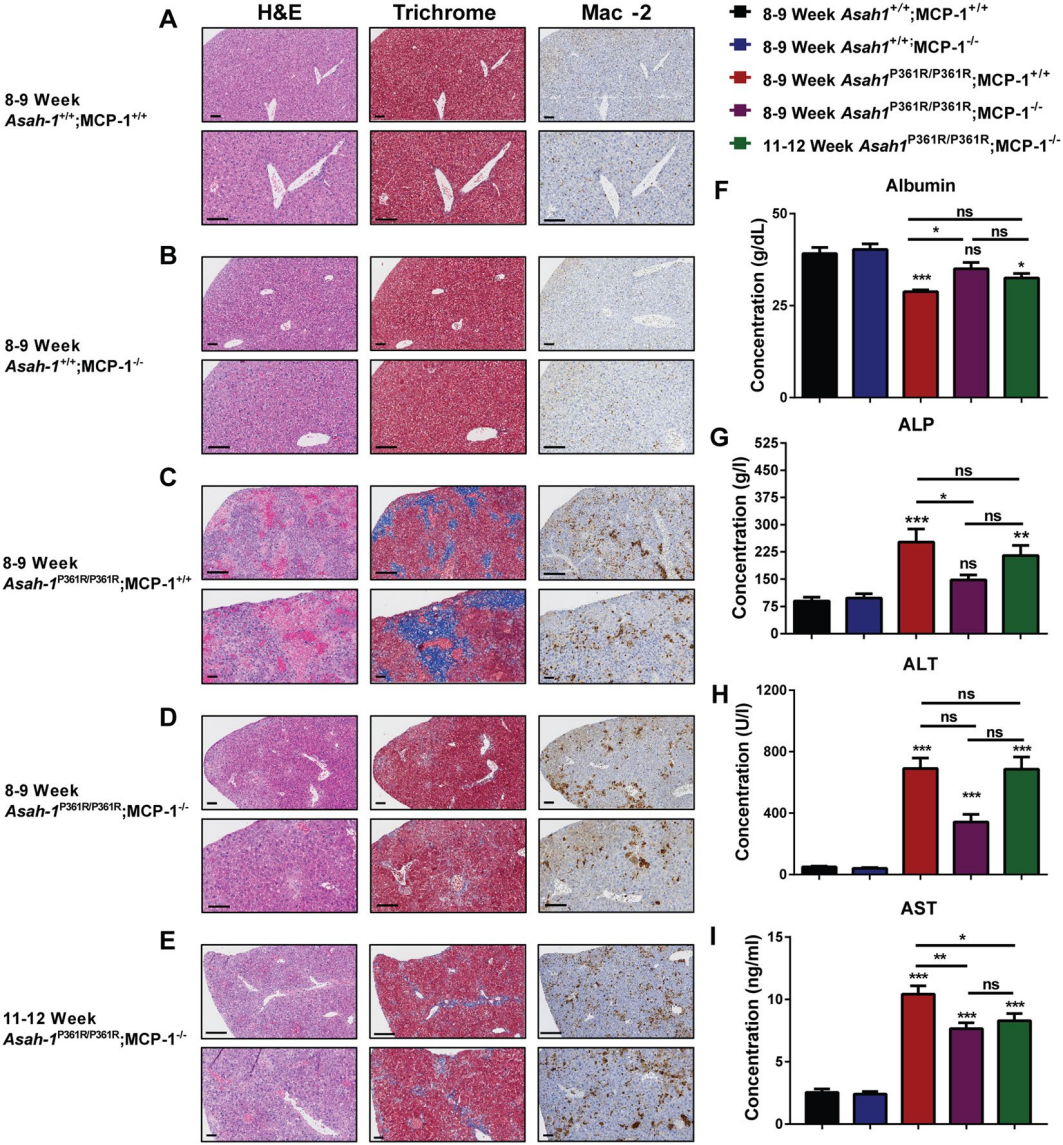
Delayed signs of liver injury and fibrosis in *Asah1*^P361R/P361R^;MCP-1^−/−^ mice. Light micrographs of liver sections stained for hematoxylin and eosin (H&E), Masson’s trichrome, and immunohistochemistry (IHC) for Mac-2 in 8–9-week-old *Asah1*^+/+^;MCP-1^+/+^ mice (**A**), 8–9-week-old *Asah1*^+/+^;MCP-1^−/−^ mice (**B**), 8–9-week-old *Asah1*^P361R/P361R^;MCP-1^+/+^ mice (**C**), 8–9-week-old *Asah1*^P361R/P361R^;MCP-1^−/−^ mice (**D**), and 11–12-week-old *Asah1*^P361R/P361R^;MCP-1^−/−^ mice (**E**). The original magnification of the top panel is 20x  where the scale bar represents 100 µm and the original magnification of the bottom panel is 40x  where the scale bar represents 50 µm. Liver function metabolites and enzymes were analyzed in serum from 8–9-week-old mice for each genotype, and samples from 11–12-week-old *Asah1*^P361R/P361R^;MCP-1^−/−^ mice. Analytes from the biochemistry panel include; Albumin (**F**), alkaline phosphatase (ALP) (**G**), alanine aminotransferase (ALT) (**H**), *n* = 7–9 mice per group. ELISA for aspartate aminotransferase (AST) (**I**). All comparisons were made between 8–9-week-old *Asah1*^P361R/P361R^;MCP-1^+/+^, 8–9-week-old *Asah1*^P361R/P361R^;MCP-1^−/−^ and 11–12-week-old *Asah1*^P361R/P361R^;MCP-1^−/−^ mice. *n* = 8 mice per group. ns (not significant), *p < 0.05, **p < 0.01,***p < 0.001.

Histological analyses (via H&E, Trichrome and IHC staining for Mac-2) demonstrated significant tissue disruption, fibrosis, and macrophage infiltration in liver tissue from 8–9-week-old *Asah1*^P361R/P361R^;MCP-1^+/+^ mice compared to controls (Fig. 4A–C). The infiltration and fibrosis present in 8–9-week-old *Asah1*^P361R/P361R^;MCP-1^+/+^ mouse livers was more pervasive compared to samples from 8–9-week-old *Asah1*^P361R/P361R^;MCP-1^−/−^ animals (Fig. 4C,D). The formation of large, foamy histiocytes and fibrotic regions increased by 11–12 weeks of age in *Asah1*^P361R/P361R^;MCP-1^−/−^ mice (Fig. 4E) but not to the extent seen in *Asah1*^P361R/P361R^;MCP-1^+/+^ mice. Albumin levels in serum showed a significant decrease in samples from *Asah1*^P361R/P361R^;MCP-1^+/+^ mice when compared to those from aged-matched controls (Fig. 4F). Ablation of MCP-1 normalized albumin levels in 8–9-week-old *Asah1*^P361R/P361R^;MCP-1^−/−^ mice, which was later lost in 11–12-week-old animals when compared to 8–9-week-old *Asah1*^+/+^;MCP-1^+/+^ mice (Fig. 4F). Although a decrease was detected in 11–12-week-old double mutants, there was no significant change when compared to 8–9-week-old *Asah1*^P361R/P361R^;MCP-1^−/−^ mice (Fig. 4F). We also measured various enzymes that are associated with liver injury. At 8–9 weeks of age, alkaline phosphatase (ALP) levels were increased in *Asah1*^P361R/P361R^;MCP-1^+/+^ mice compared to age-matched controls (Fig. 4G). ALP from 8–9-week-old *Asah1*^P361R/P361R^;MCP-1^−/−^ mice was not different from age-matched controls, but 11–12-week-old *Asah1*^P361R/P361R^;MCP-1^−/−^ mice did display elevated ALP, similar to the levels seen in 8–9-week-old *Asah1*^P361R/P361R^;MCP-1^+/+^ mice (Fig. 4G). In comparison to control mice, alanine aminotransferase (ALT) was elevated similarly in 8–9-week-old *Asah1*^P361R/P361R^;MCP-1^+/+^ mice and in both 8–9 and 11–12-week-old *Asah1*^P361R/P361R^;MCP-1^−/−^ mice (Fig. 4H). In comparison to control mice, aspartate aminotransferase (AST) levels were elevated in both 8–9 and 11–12-week-old *Asah1*^P361R/P361R^; MCP-1^−/−^ mouse samples but not to the same extent as that seen in samples from 8–9-week-old *Asah1*^P361R/P361R^;MCP-1^+/+^ mice (Fig. 4I).

### Reduction of pulmonary infiltrates and protein accumulation

**Figure 5:**
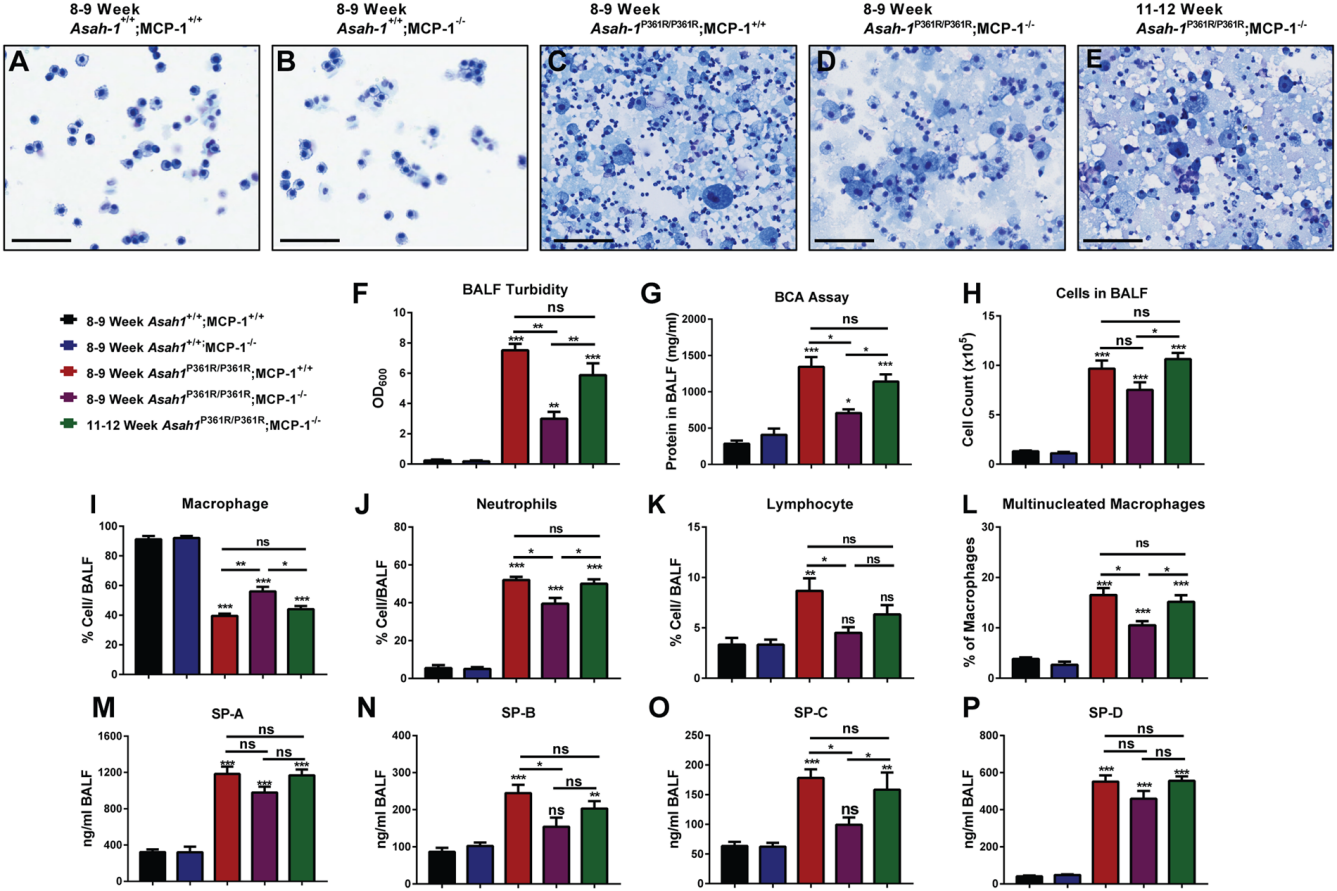
Bronchial alveolar lavage fluid (BALF) from *Asah1*^P361R/P361R^;MCP-1^−/−^ mice lungs displays lessened signs of pulmonary inflammation and surfactant protein accumulation. Representative light micrographs of Cytospin BALF slides from 8–9-week-old mice in all genotypes and 11–12-week-old from *Asah1*^P361R/P361R^; MCP-1^−/−^ mice. The original magnification was set at 20x and the scale bar represents 100 µm at equal 1:2 dilutions (**A**–**E**). BALF supernatant from 8–9-week-old mice in all genotypes and 11–12-week-old from *Asah1*^P361R/P361R^;MCP-1^−/−^ mice measuring turbidity by absorbance at 600 nm (**F**) and protein concentration from Bicinchoninic acid (BCA) assay (**G**) *n* = 5–6. Cell counts and differentials were performed on BALF cell pellets from 8–9-week-old mice in all genotypes and from 11–12-week-old *Asah1*^P361R/P361R^;MCP-1^−/−^ mice. Kwik-Diff stained cytospin slides show BALF cell counts (**H**), macrophages (**I**), neutrophils (**J**), lymphocytes (**K**), and percentage of multinucleated macrophages out of total macrophages (**L**) *n* = 5. ELISAs were performed on BALF supernatant from 8–9-week-old mice in all genotypes and 11–12-week-old *Asah1*^P361R/P361R^;MCP-1^−/−^ mice for SP-A (**M**), SP-B (**N**), SP-C (**O**) and SP-D (**P**) *n* = 5–6. All comparisons were made between 8–9-week-old *Asah1*^+/+^;MCP-1^+/+^, *Asah1*^P361R/P361R^;MCP-1^+/+^, *Asah1*^P361R/P361R^;MCP-1^−/−^ and 11–12-week-old *Asah1*^P361R/P361R^;MCP-1^−/−^ mice. ns (not significant), *p < 0.05, **p < 0.01, ***p < 0.001.

Recently we demonstrated that *Asah1*^P361R/P361R^;MCP-1^+/+^ mice develop chronic lung injury and inflammation^17^. Here we examined the effects of deletion of MCP-1 on those parameters. Micrographs of representative BALF Cytospin slides showed significant cellularity and proteinaceous material in samples from both 8–9-week-old *Asah1*^P361R/P361R^;MCP-1^+/+^ and 8–9-week-old *Asah1*^P361R/P361R^;MCP-1^−/−^ mice in comparison to controls (Fig. 5A–D). That said, samples from 8–9-week-old *Asah1*^P361R/P361R^;MCP-1^−/−^ mice demonstrated a reduction in infiltrating cells and a lesser abundance of extracellular protein-stained debris than samples from the 8–9-week-old *Asah1*^P361R/P361R^;MCP-1^+/+^ mice (Fig. 5D). That latter phenotype became even more pronounced in the 11–12 week *Asah1*^P361R/P361R^;MCP-1^−/−^ samples (Fig. 5E). This observation was also reflected in BALF supernatant turbidity and protein concentration analyses. At 8–9 weeks of age, BALF turbidity and protein levels from *Asah1*^P361R/P361R^;MCP-1^−/−^ were significantly lower than in 8–9-week-old *Asah1*^P361R/P361R^;MCP-1^+/+^ mice (Fig. 5F–G). However, by 11–12 weeks of age, BALF turbidity and protein levels from *Asah1*^P361R/P361R^;MCP-1^−/−^ mice had increased to levels similar to 8–9-week-old *Asah1*^P361R/P361R^;MCP-1^+/+^ mice (Fig. 5F–G). Quantitation of BALF cells was performed on Kwik-Diff stained Cytospin slides. Total cell counts in samples from 8–9-week-old *Asah1*^P361R/P361R^;MCP-1^−/−^ mice were not significantly different from samples from age-matched *Asah1*^P361R/P361R^;MCP-1^+/+^ mice, but the *Asah1*^P361R/P361R^;MCP-1^+/+^ mice did display a significant increase in cell recruitment between 8–9 and 11–12 weeks of age (Fig. 5H). BALF cell differentials from 8–9-week-old *Asah1*^P361R/P361R^;MCP-1^−/−^ mice revealed an increase in macrophage abundance and reductions in neutrophil, lymphocyte, and percentage of multinucleated macrophages when compared to age-matched *Asah1*^P361R/P361R^;MCP-1^+/+^ mice (Fig. 5I–L). The percentage of total macrophage, neutrophil, and percentage of multinucleated macrophages in the BALF of 11–12-week-old *Asah1*^P361R/P361R^;MCP-1^−/−^ mice had normalized to the levels found in 8–9-week-old *Asah1*^P361R/P361R^;MCP-1^+/+^ mice (Fig. 5I–L). Thus, it appears that deletion of MCP-1 may impede cellular recruitment to the lungs. This trend was also observed when an ELISA was performed on BALF supernatant to detect surfactant proteins (SP). There we observed a reduction of detectable SP-B and SP-C in samples from 8–9-week-old *Asah1*^P361R/P361R^;MCP-1^−/−^ mice. By 11–12 weeks of age SP-B and SP-C in the BALF of *Asah1*^P361R/P361R^;MCP-1^−/−^ mice were detected at levels similar to 8–9-week-old *Asah1*^P361R/P361R^;MCP-1^+/+^ mice (Fig. 5N and O). Interestingly, though elevated in BALF supernatants from all *Asah1*^P361R/P361R^ mice, no significant changes were seen in Surfactant Protein (SP) A or D levels with altered MCP-1 expression (Fig. 5M and P).

### Brain and behavioral phenotypes persist in MCP-1 deficient mice

**Figure 6:**
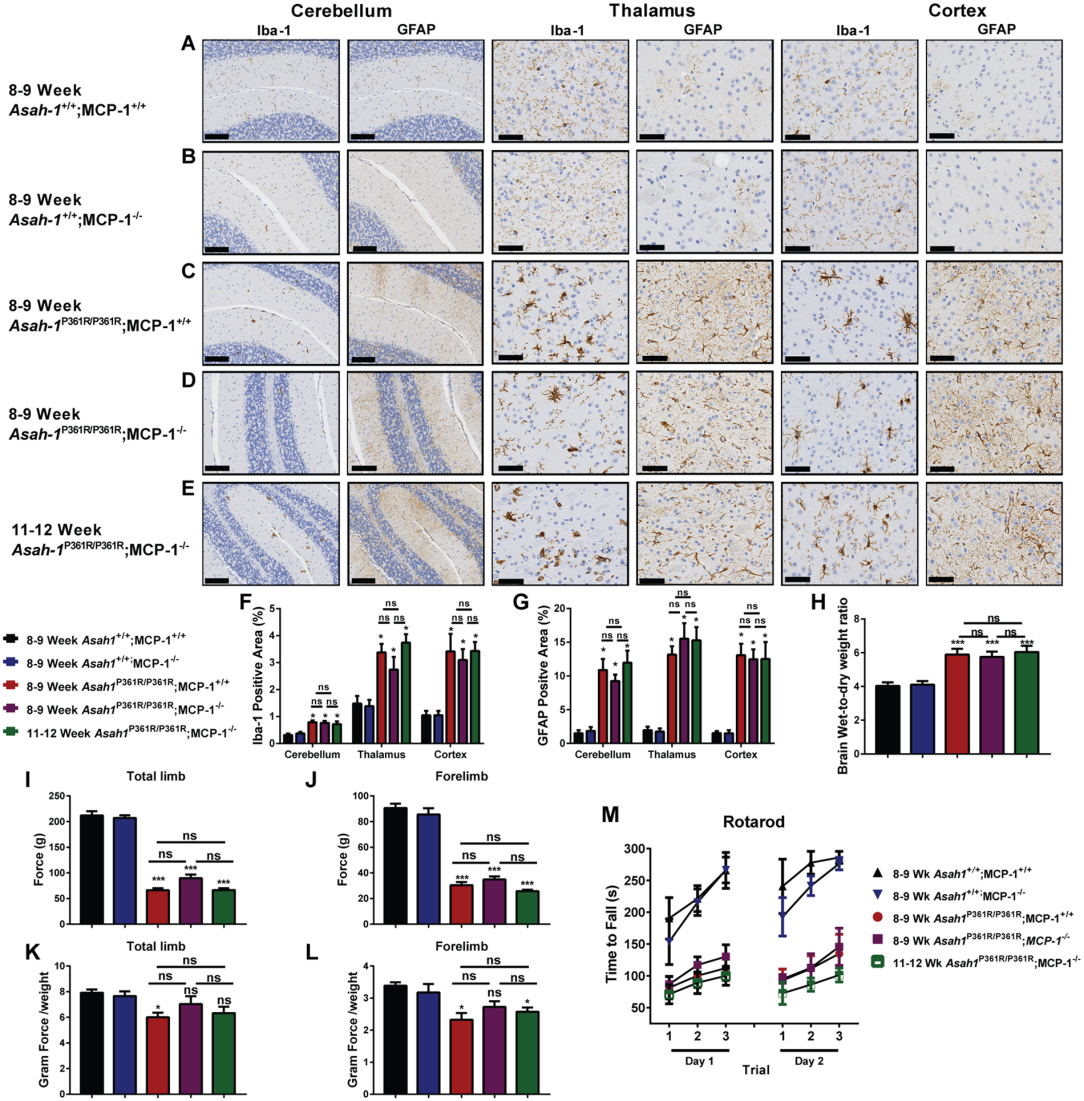
Brain and behavioral deficits persist in *Asah1*^P361R/P361R^;MCP-1^−/−^ mice. Representative micrographs from 8–9-week-old and 11–12-week-old mice from all genotypes featuring cerebellum, thalamus, and cortex regions stained for GFAP and Iba-1. Original magnification of cerebellum samples is 10x. The scale bar represents 100 µm and the original magnifications of the thalamus and cortex samples were 20x  wherein the scale bar represents 50 µm (**A**–**E**). The percentage of GFAP and Iba-1 positive staining from the cerebellum, thalamus, and cortex regions. Quantitation was performed on micrographs from 8–9 and 11–12-week-old mice. *n* = 3–4 mice per genotype (**F** and **G**). Brain wet-to-dry weight ratios were performed on 8–9 and 11–12-week-old mice. *n* = 3–4 mice per genotype (**H**). Behavioral tests were performed on 8–9 and 11–12-week-old mice for grip strength (**I**–**L**) and Rotarod proficiency (**M**). *n* = 8–10 per genotype. ns (not significant), *p < 0.05 **p < 0.01, ***p < 0.001.

*Asah1*^P361R/P361R^;MCP-1^+/+^ mice develop significant central nervous system defects including neuroinflammation, demyelination, neurodegeneration, and hydrocephaly. These become progressively more severe as the disease manifests fully^18^. To assess the state of neuroinflammation, brain tissues were stained with GFAP and Iba-1 to highlight activated astrocytes and microglia. Brain samples from 8–9-week-old *Asah1*^P361R/P361R^;MCP-1^+/+^ and both 8–9 and 11–12-week-old *Asah1*^P361R/P361R^;MCP-1^−/−^ mice have similar Iba-1 and GFAP staining patterns in the cerebellum, thalamus, and cerebral cortex (Fig. 6A–G). This may indicate that there is no additive effect of inflammation between 8–9 and 11–12 weeks of age in these models. From brain wet-to-dry weight ratios, no changes in brain edema were found between 8–9-week-old *Asah1*^P361R/P361R^*;MCP*-1^+/+^ and both 8–9 and 11–12-week-old *Asah1*^P361R/P361R^;MCP-1^−/−^ mice (Fig. 6H).

### Behavioral phenotypes unchanged in MCP-1 deficient mice

The *Asah1*^P361R/P361R^;MCP-1^+/+^ mice replicate behavioral features that are typically present in FD patients^18^. Amongst these the *Asah1*^P361R/P361R^;MCP-1^+/+^ mice have shown decreased locomotion, decreased exploratory behavior, increased thigmotaxis, and weakness in grip strength and rotarod testing^18^. To assess whether MCP-1 ablation may improve behavior parameters, we repeated the grip strength and Rotarod tests. In the grip strength test, both 8–9 and 11–12 week-old *Asah1*^P361R/P361R^;MCP-1^−/−^ mice displayed significantly decreased ability on both total limb and forelimb strength tests when compared to age-matched control mice (Fig. 6I and J). However, when we normalize for body weight, 8–9 week-old *Asah1*^P361R/P361R^;MCP-1^−/−^ mice showed no differences in total limb or forelimb strength compared to age-matched controls (Fig. 6K,L). At 11–12 weeks, *Asah1*^P361R/P361R^;MCP-1^−/−^ mice lost their total limb strength but maintained a hind-limb strength advantage (Fig. K,L). This may suggest that hind-limb weakness occurs prior to forelimb weakness in our model. However, due to their tight skin and our subsequent difficulty in scruffing *Asah1*^P361R/P361R^;MCP-1^+/+^ and *Asah1*^P361R/P361R^;MCP-1^−/−^ mice, we could not reliably obtain hind-limb data. Lastly, the Rotarod test demonstrated that 8–9 and 11–12 week-old *Asah1*^P361R/P361R^;MCP-1^−/−^ animals displayed a motor and endurance phenotype that largely mirrors what we reported in 8–9-week-old *Asah1*^P361R/P361R^;MCP-1^+/+^ mice^18^ (Fig. 6M). Taken together, there appears to be no improvement in neuroinflammation or brain edema, and little improvement in age-matched motor strength, in the test animals.

### Tissue-specific changes in sphingolipid profiles due to MCP-1 deletion

**Figure 7:**
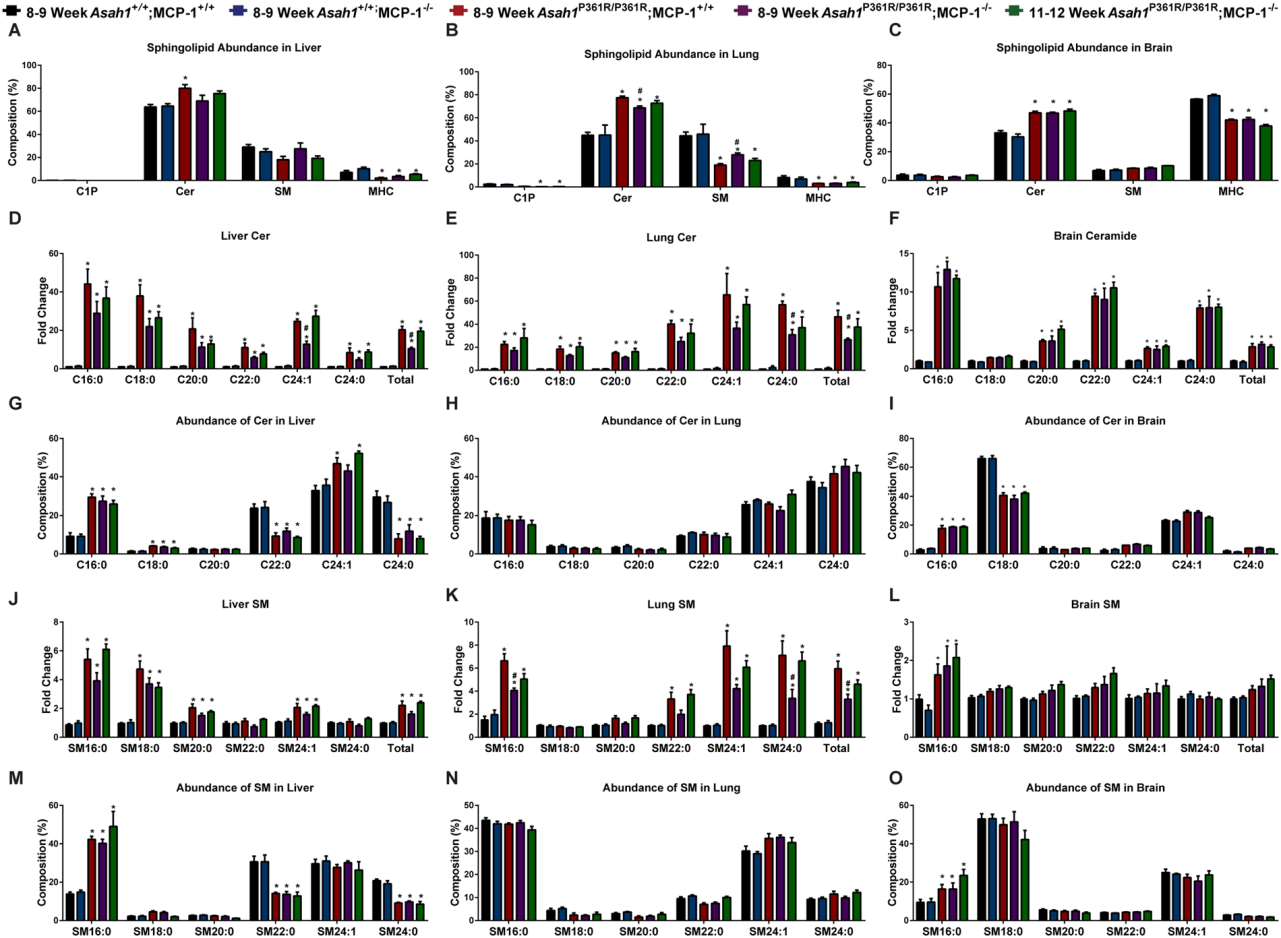
Sphingolipid accumulation and abundance changes are organ specific in the *Asah1*^P361R/P361R^;MCP-1^−/−^ mice. Percentage distribution of all total sphingolipids measured in liver, lung, and brain tissue lysates (**A**–**C**). Ceramide (Cer) species in liver, lung, and brain (**D**–**F**). Relative abundance of Cer species in liver, lung, and brain (**G**–**I**). Sphingomyelin (SM) species in liver, lung, and brain (**J**–**L**) and relative abundance of SM species in liver, lung, and brain (**M**–**O**). *n* = 4–6 per genotype. All comparisons were made between samples from 8–9-week-old *Asah1*^P361R/P361R^;MCP-1^+/+^, 8–9-week-old *Asah1*^P361R/P361R^;MCP-1^−/−^ and 11–12-week-old *Asah1*^P361R/P361R^;MCP-1^−/−^ mice. ns (not significant), *Represents post-hoc test compared to *Asah1*^+/+^;MCP-1^+/+^ *p < 0.05, **p < 0.01,***p < 0.001. ^#^Represents post-hoc test compared to *Asah1*^P361R/P361R^;MCP-1^+/+ *#*^*p* < 0.05.

Sphingolipid profiles were measured via liquid chromatography-mass spectrometry (LC-MS) in lipid extracts from liver, lung, and brain tissue lysates from control and test animals. Sphingolipids assayed included ceramides (Cer), ceramide-1-phosphate (C1P), sphingomyelin (SM), monohexosylceramides (MHC), sphingosine (Sph), and sphingosine-1-phosphate (S1P). The most abundant sphingolipid in all three tissues in 8–9-week-old *Asah1*^P361R/P361R^*;MCP*-1^+/+^ mice was Cer (Fig. 7A–C and Supplementary Fig S6). The greatest changes in abundance were in the lung, where 8–9-week-old *Asah1*^P361R/P361R^;MCP-1^−/−^ mice displayed increased Cer and SM levels compared to control mice, but had a significant reduction in Cer and increased SM when compared to 8–9-week-old *Asah1*^P361R/P361R^;MCP-1^+/+^ mice (Fig. 7B). Interestingly, this shift was no longer present in tissues from 11–12-week-old *Asah1*^P361R/P361R^;MCP-1^−/−^ mice. When we examined liver tissues, 8–9-week-old *Asah1*^P361R/P361R^*;MCP*-1^−/−^ mice had a reduction in total and C24:1 Cer compared to samples from 8–9-week-old *Asah1*^P361R/P361R^;MCP-1^+/+^ mice. By 12 weeks of age, the C24:1 and total Cer content in liver were increased to the same level as that seen in 8–9-week-old *Asah1*^P361R/P361R^;MCP-1^+/+^ *mice*. There were no differences between *Asah1*^P361R/P361R^*;MCP*-1^+/+^ *and Asah1*^P361R/P361R^;MCP-1^−/−^ mice for SM, MHCs, C1P, and S1P for the liver (Fig. 7J,M and Supplementary Figs S5 and S6). Sph in liver was unchanged between *Asah1*^P361R/P361R^;MCP-1^−/−^ mice and age-matched controls, however a decrease was found when compared to the 11–12-week-old group (Supplementary Fig. S6A).

The greatest changes in Cer levels were seen in the lungs. There we observed a reduction in C24:1, C24:0, and total Cer content in samples from 8–9-week-old *Asah1*^P361R/P361R^;MCP-1^−/−^ animals compared to those from age-matched *Asah1*^P361R/P361R^;MCP-1^+/+^ mice (Fig. 7E). This relationship is reflected in a significant increase in SM24:1, SM24:0, and total SMs between samples from 8–9-week-old *Asah1*^P361R/P361R^;MCP-1^−/−^ animals compared to those from age-matched *Asah1*^P361R/P361R^;MCP-1^+/+^ mice (Fig. 7K). In the lungs, we observed a significant reduction in C24:0 MHCs, total MHCs, C24:0 C1P, and total C1P species but no changes in Sph and S1P between samples from 8–9 and 11–12-week-old *Asah1*^P361R/P361R^;MCP-1^−/−^ animals compared to those from 9-week-old *Asah1*^P361R/P361R^;MCP-1^+/+^ mice (Supplementary Figs S5 and S6). In brain lysates, there were no observed changes in the sphingolipid species we queried between samples from 8–9 and 11–12-week-old *Asah1*^P361R/P361R^;MCP-1^−/−^ animals compared to those from 9-week-old *Asah1*^P361R/P361R^;MCP-1^+/+^ mice.

### Unique Cytokine signature in *Asah1*^P361R/P361R^;MCP-1^−/−^ double mutants

**Figure 8:**
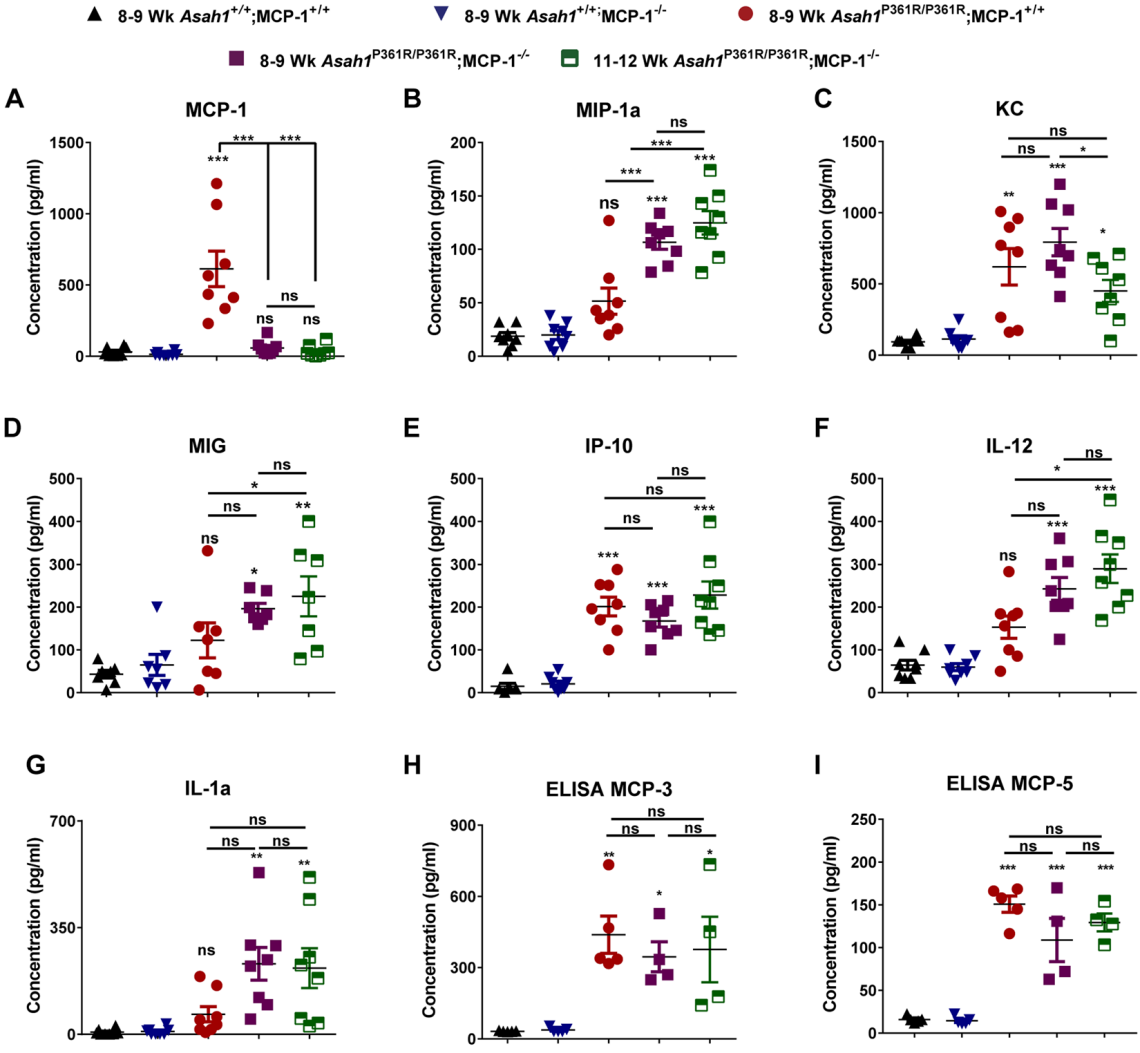
Altered cytokines in the serum of *Asah1*^P361R/P361R^;MCP-1^−/−^ mice. Cytokines were measured in serum of 8–9-week-old mice for each genotype and 11–12-week-old *Asah1*^P361R/P361R^;MCP-1^−/−^ mice. Levels of CCL2 (MCP-1) (**A**), CCL3 (MIP-1α) (**B**), keratinocyte chemoattractant (KC) (**C**), CXCL9 (MIG) (D), CXCL10 (IP-10) (**E**), interleukin-12 (IL-12) (**F**), and interleukin-1-alpha (IL-1α) (**G**) were measured. *n* = 8. Levels of CCL7 (MCP-3) (**H**) and CCL12 (MCP-5) (**I**) measured with ELISA. *n* = 4–5. All comparisons were made between 8–9-week-old *Asah1*^+/+^;MCP-1^+/+^, *Asah1*^P361R/P361R^;MCP-1^+/+^, *Asah1*^P361R/P361R^;MCP-1^−/−^ and 11–12-week-old *Asah1*^P361R/P361R^;MCP-1^−/−^ mice. ns (not significant), *p < 0.05, **p < 0.01, ***p < 0.001.

To access whether the absence of MCP-1 may affect cytokine profiles, a multiplex mouse cytokine panel was queried using serum samples. As we have seen before, MCP-1 levels were found to be dramatically increased in 8–9-week-old *Asah1*^P361R/P361R^;MCP-1^+/+^ mice^4^. As expected, no MCP-1 was found in serum samples from 8–9 and 11–12 week-old *Asah1*^P361R/P361R^;MCP-1^−/−^ mice (Fig. 8A). Interestingly, the levels of monocyte inflammatory protein-1 alpha (MIP-1α) in samples from 8–9-week-old *Asah1*^P361R/P361R^;MCP-1^−/−^ mice was significantly higher compared to those obtained from age-matched *Asah1*^P361R/P361R^;MCP-1^+/+^ mice; these values were even further increased in samples from such mice at 11–12 weeks of age (Fig. 8B). Keratinocyte chemoattractant (KC) concentrations peaked in samples from both 8–9-week-old *Asah1*^P361R/P361R^;MCP-1^+/+^ and *Asah1*^P361R/P361R^;MCP-1^−/−^ mice. While KC levels were detected at lower levels in 11–12-week-old samples compared to 8–9-week-old samples in *Asah1*^P361R/P361R^;MCP-1^+/+^, they were nonetheless statistically elevated when compared to controls (Fig. 8C). Interferon gamma-induced protein 10 (IP-10) concentrations in serum from 8–9 and 11–12-week-old *Asah1*^P361R/P361R^;MCP-1^−/−^ mice were elevated to similar levels as those seen in samples from 8–9-week-old *Asah1*^P361R/P361R^;MCP-1^+/+^ mice (Fig. 8E). Interestingly, monocyte induced by gamma interferon (MIG), Interleukin (IL) IL-12, and IL1α were not significantly increased in samples from 8–9-week-old *Asah1*^P361R/P361R^; MCP-1^+/+^ mice when compared to control mice but were indeed significantly increased in samples from 8–9-week-old *Asah1*^P361R/P361R^;MCP-1^−/−^ mice (Fig. 4D,F,G). Although not statistically significant, IL1β, and IL13 concentrations displayed a trend towards an increase in 11–12-week-old *Asah1*^P361R/P361R^*;MCP*-1^−/−^ samples (Supplementary Fig. S7). No differences were seen for basic fibroblast growth factor (bFGF), IL-4, IL-5, IL-6, IL-10, IL-17, granulocyte macrophage colony-stimulating factor (GM-CSF), interferon gamma (IFNγ), or vascular endothelial growth factor (VEGF) (Supplementary Fig. S7). We further measured MCP-3 and MCP-5 levels by ELISA in mouse serum. Both MCP-3 and MCP-5 levels were significantly elevated in 8–9-week-old *Asah1*^P361R/P361R^; MCP-1^+/+^ when compared to age-matched controls (Fig. 8H,I). MCP-3 and MCP-5 levels were also elevated in both 8–9 and 11–12-week-old *Asah1*^P361R/P361R^;MCP-1^−/−^ mice. No differences in MCP-3 and MCP-5 levels between samples from 8–9-week-old *Asah1*^P361R/P361R^;MCP-1^+/+^, 8–9 and 11–12 week-old *Asah1*^P361R/P361R^;MCP-1^−/−^ mice were observed.

## Discussion

The signs and symptoms of FD can manifest along a spectrum. In its moderate form, FD patients will develop arthritis-like symptoms such as joint pain and nodule formation^3^. In its severe classical form, however, FD patients will often have significant inflammation, neurological complications, and respiratory failure^1^. In our FD mouse, widespread inflammation has been reported to affect many organs in the *Asah1*^P361R/P361R^;MCP-1^+/+^ mouse^4,12,17^. Here we examine multiple parameters of FD manifestations in the context of deletion of a key chemokine (MCP-1) that we identified previously as a biomarker for this disorder^6^. Our analyses of the hematopoietic, hepatic, pulmonary, and neurological systems demonstrates that the severity of MCP-1-mediated inflammation is organ dependent. Our results here further demonstrate that attenuation of MCP-1 in FD mice can affect ceramide accumulation, improve survival, and delay pathology.

The *Asah1*^P361R/P361^;MCP-1^+/+^ mouse has impaired hematopoiesis characterized by a decline in pre- and pro-B cell numbers in the bone marrow of aged mice and a reduction of CD4^+^ CD8^+^ T-cells within the thymus of 5-weeks-of-age animals that progressively worsens^12^. Despite increased longevity and a delay in weight loss and skin elasticity at 8–9-weeks-of-age, the *Asah1*^P361R/P361R^;MCP-1^−/−^ mice share a similar hematological profile to the *Asah1*^P361R/P361R^;MCP-1^+/+^ mice. Histological analyses revealed tissue damage to the architecture of the bone marrow, thymus, and spleen with 11–12 weeks of age *Asah1*^P361R/P361R^;MCP-1^−/−^ mice, sharing a profile that is very similar to 8–9-week-old *Asah1*^P361R/P361R^;MCP-1^+/+^ mice. Since age-matched *Asah1*^P361R/P361R^;MCP-1^−/−^ mice show reduced leukocytosis and a reduction in granulocytes in the spleen, it suggests that absence of MCP-1 can slow the rate of macrophage infiltration; this also suggests, however, that the bone marrow and thymus may be more sensitive to inflammation and injury when the sphingolipid balance is perturbed. This phenotype has also been noted in studies on the sphingosine 1-phosphate lyase deficient mouse (SGPL^−/−^), where investigators showed impaired B-cell development in the bone marrow, thymic atrophy associated with a reduction in CD4^+^ and CD8^+^ thymocytes, and an increase in thymic ceramide levels^19,20^.

Deletion of MCP-1 also leads to improvement in the hepatic system. In comparison to age-matched control animals, there was a reduction in liver injury enzymes, decreased formation of characteristic large foamy macrophages, and less tissue fibrosis in the double mutants. MCP-1 expression within the liver has been shown to play a role in hepatic inflammation and fibrosis in patients with hepatitis C^21^. Similarly, inhibition of MCP-1 with the structured L-enantiomeric RNA oligonucleotide mNOX-E36 (aka Spiegelmer) has shown promise in reducing fibrosis in murine models of chronic liver disease^22^. Since we also see a reduction in accumulated ceramides, it is possible that, similar to the lungs, an absence of MCP-1 is reducing the rate of recruitment of cells which is reducing the total amounts of ceramide as well as the inflammation-induced injury that we normally observe in *Asah1*^P361R/P361R^;MCP-1^+/+^ mice.

Infiltration of immune cells into the lungs and respiratory failure can be a significant problem in FD^1^. Recently we have shown that *Asah1*^P361R/P361R^;MCP-1^+/+^ mice develop chronic lung injury caused by a combination of inflammation and vascular leakage^17^. Here we find that MCP-1 deletion results in significant improvement of lung phenotypes. BALF from *Asah1*^P361R/P361R^;MCP-1^−/−^ mice revealed both lower protein content and lower cell counts than that from *Asah1*^P361R/P361R^;MCP-1^+/+^ mice. In addition, there was a mild reduction in lipo-protein-like material in the BALF, which also correlated with a reduction in various surfactant proteins. Surprisingly, in addition to reduced inflammation in the lungs, we also observed a reduction in all measured sphingolipids in lung tissue lysates (Fig. 8B). One explanation for this may be the reduced recruitment of cells within the lung parenchyma. Another explanation may be the reduced inflammatory cues produced by MCP-1 localized within the lungs; the role of MCP-1 as an instigator of inflammation and as a potential biomarker has been widely reported^23,24^. Increased MCP-1 can also be detected in rare diseases like pulmonary alveolar proteinosis, chronic obstructive pulmonary disease, as well as in severe cases of acute lung injury^25–27^. In addition, use of anti-rat MCP-1 antibodies has shown efficacy in reducing not only the size, but the quantity of glucan-induced pulmonary granulomas vasculitis in rats^28^. Due to the widespread role of MCP-1 in lung pathology, it is thus possible that in the context of FD, MCP-1-mediated recruitment of inflammatory cells may be more penetrant and pernicious in the lung than other organs in our mouse model.

The brains of *Asah1*^P361R/P361R^;MCP-1^+/+^ mice show an accumulation and a shift in the relative abundance of various sphingolipids, the presence of cellular storage bodies in various neuronal cells, signs of inflammation, and elevated MCP-1 levels^4,18^. Histological analyses on age-matched brains from *Asah1*^P361R/P361R^;MCP-1^−/−^ mice also demonstrated significant astrocytosis and large activated microglia (Fig. 6D,E). Though not statistically significant, there also appears to be a trend towards an increase in percentage staining of Iba-1 from 8–9-week-old to 11–12-week-old brains of *Asah1*^P361R/P361R^;MCP-1^−/−^ mice. Behavioral assays on the *Asah1*^P361R/P361R^;MCP-1^−/−^ mice demonstrated little change from controls, however we do show a mild improvement in forelimb grip strength force when normalized for body weight (Fig. 6J,L). Together, this demonstrates that deletion in MCP-1 may have a minor effect in delaying the signs of neural inflammation in our Farber mice.

Inhibition of inflammatory cytokines has shown variable results in LSD brain models. For example, in the Hexβ^−/−^; Mip-1α^−/−^ mouse, deletion of Mip-1α led to an improvement in brain pathology^29^. The opposite outcome was observed when the same cross was performed with the Niemann-Pick Type C (NPC^−/−^) mouse model^30^. Both of those LSD models demonstrate some shared phenotypes; however, in the latter cross the mice experienced a decreased lifespan^29,30^. The group that characterized this outcome rationalized that in the context of NPC, it is possible that inflammation in the brain may serve a protective role^30^. This theme is also shared in the case of globoid cell leukodystrophy (GLD; Krabbe’s disease), which shares some neurological phenotypes with the Farber mouse such as activated microglia/macrophages and demyelination^18^. To genetically remove macrophages, one group crossed the GLD Twitcher (*twi*^−/−^) mouse to the macrophage-deficient osteoporotic (CSF-1^op/op^) mouse^31^. Double mutants there not only had a shorter lifespan but also manifested an exacerbated neurological phenotype compared to control *twi*^−/−^ mice^31^.

Brain sphingolipid profiles of our *Asah1*^P361R/P361R^;MCP-1^−/−^ mice were largely unchanged compared to *Asah1*^P361R/P361R^;MCP-1^+/+^ mice. In our analyses of Sph there was a minor but significant reduction in Sph for both the *Asah1*^P361R/P361R^;MCP-1^+/+^, and *Asah1*^P361R/P361R^;MCP-1^−/−^ groups. This observation deviates somewhat from our previously published results wherein we detected a modest increase in Sph in brain lysates from ACDase deficient mice^18^. The variability could be a combination of the different tissue homogenization protocols, lipid extraction methods, internal standards used, instrumentation, and LC/MS protocols as these were processed at different sites. Interestingly this contrasting result may be exclusive to Sph, however, as all other sphingolipids measured in both studies displayed the same trend^18^.

Nonetheless, elevated ceramide levels were noted in the brain. Increases in ceramides are also detected in various CNS conditions, such as in multiple sclerosis (MS)^32^. This study showed that activated astrocytes that were preferably localized to MS-related brain lesions were the main contributors of the increased levels of ceramides detected^32^. Furthermore, inhibition of the *de novo* pathway of ceramide synthesis with the S1P analogue FTY720 was able to attenuate reactive phenotype of astrocytes^32^. Therefore, it is possible that the inflammatory consequences of increased MCP-1 in the brain are masked by the deleterious effects of significant ceramide and sphingolipid accumulation

Our study demonstrated that genetic ablation of MCP-1 allowed *Asah1*^P361R/P361R^ mice to survive up to 14 weeks of age. This increased longevity coincided with reduced pathology in the lungs and liver of 8–9-week-old *Asah1*^P361R/P361R^;MCP-1^−/−^ and age matched controls. By 11–12-weeks of age the disease phenotypes in *Asah1*^P361R/P361R^;MCP-1^−/−^ mice largely mirrors that of 8–9-week-old *Asah1* ^P361R/P361R^;MCP-1^+/+^ mice. While little to no changes were seen in the hematopoietic and neurological systems, the absence of MCP-1 slowed the deleterious effects of ceramide-induced inflammation. In addition, we found new changes in the cytokine profiles that may, in part, explain the impeded pathology in the *Asah1*^P361R/P361R^;MCP-1^−/−^ mice.

KC expression was previously found to peak at 7–8 weeks and decline by 9 weeks in *Asah1*^P361R/P361R^;MCP-1^+/+^ mice^6^. In this study, deletion of MCP-1 caused a shift where KC was seen to peak at 8–9-weeks and later decrease in 11–12 week samples (Fig. 8C). Further, our data also highlights a possible compensatory accumulation of the cytokines MIP-1α, MIG, IL-12, and IL-1α in the double mutant animals. Along these lines, we previously showed that MIP-1α levels increase by 5 weeks and peaked at 9 weeks of age in *Asah1*^P361R/P361R^;MCP-1^+/+^ mice^6^. In this study, we found no statistical significance for MIP-1α in 8–9-week-old *Asah1*^P361R/P361R^;MCP-1^+/+^ mice samples. This may in part be due to sample variability in the *Asah1*^P361R/P361R^;MCP-1^+/+^ samples. However MIP-1α was elevated and significant in both 8–9 and 11–12-week-old *Asah1*^P361R/P361^;MCP-1^−/−^ mice samples. MIP-1α, a well-studied chemokine, is known for its roles in macrophage recruitment and inflammatory responses. In fact, other studies of LSDs such as Gaucher disease and Sandhoff’s disease have reported elevations of MIP-1α as part of disease progression in patient and mouse models^29,33^. In Gaucher disease, where glucosylceramide accumulates, infiltration of tissues and formation of large foamy macrophages is also widely abundant^34,35^. Both MCP-1 and MIP-1α have been found to be elevated in the serum samples of Gaucher patients^36^. Large foamy macrophages are a shared phenotype present in both Farber and Gaucher disease. Due to this similarity and the tight balance of sphingolipid metabolism, the compensatory accumulation of MIP-1α may yet be another shared sign. While more work is required, it is possible that the inflammation observed in both Farber disease and Gaucher disease manifest similarly.

Here we also report elevations in MIG in the serum of our *Asah1*^P361R/P361^;MCP-1^−/−^ mice. In contrary, MIG from *Asah1*^P361R/P361^;MCP-1^+/+^ mice samples were not found to be statistically significant in this study but appear to show a trend towards an increase, which is also mirrored in FD patient plasma^6^. Similar findings were noted for IL-12. Lastly, IL-1α was elevated in the double mutants. This was unexpected as IL-1α in our previous data was variably expressed in mouse plasma^6^. There is evidence that activation of IL-1 may lead to the accumulation of ceramide and expression of the inflammatory enzyme COX-2 along with prostaglandin E_2_ production^37^. More work will be required to elucidate the roles of these cytokines in FD, however these data together demonstrate the complexity and nuanced cytokine/chemokine regulation that might be involved in the inflammatory response seen in FD.

Analyses of MCP-3 and MCP-5 by ELISA in mouse serum revealed significantly increased levels of these factors in samples from 8–9-week-old *Asah1*^P361R/P361^;MCP-1^+/+^ mice. This observation demonstrates that other agonists of CCR2 are also contributors to the inflammatory phenotype seen in ACDase deficiency^38,39^. While the MCP-3 and MCP-5 levels were not statistically significant between 8–9 and 11–12-weeks in the *Asah1*^P361R/P361^;MCP-1^−/−^ mice, there appeared to be a trend towards an increase. The high levels of MCP-3 and MCP-5 would partly explain the continued presence of foamy macrophages in *Asah1*^P361R/P361^;MCP-1^−/−^ mice. A previous study has shown that MCP-3^−/−^ mice that were exposed to *Listeria monocytogenes* infection showed a similar inflammatory phenotype as those of MCP-1^−/−^ mice^40^. That study demonstrated that both MCP-1 and MCP-3 act in parallel on CCR2 in the context of innate immune defense^40^. This phenomenon may apply in our model as the ablation of MCP-1 alone provided a modest lifespan improvement to *Asah1*^P361R/P361^ mice. Furthermore, another chemokine, MCP-4, which was not measured in this study, has also been shown to be an agonist for CCR2^41^. Out of all three chemokines measured, MCP-1 was detected at the highest level in serum samples of *Asah1*^P361R/P361^;MCP-1^+/+^ mice, suggesting a dominant role for that factor in macrophage recruitment in ACDase deficiency. While additional work will be required, the discovery of elevated MCP-3 and MCP-5 further underscores the importance of CCR2 signaling in ACDase biology.

MCP-1 is a potent chemo-attractant that has been implicated in exacerbating the pathology of an array of diseases. Genetic crosses between the MCP-1^−/−^ mouse and other disease models ranging from the Charcot-Marie-Tooth mouse model to the more common atherosclerosis mice models have been previously performed^42–44^. Results showed that the absence of MCP-1 led to attenuation of neuroinflammation in the case of the Charcot-Marie-Tooth model, and reduction in aortic lesions the atherosclerotic ApoE deficient mouse^42,44^.

MCP-1 has also been identified as a therapeutic target for other conditions including kidney and heart disease^45,46^. Use of the structured L-enantiomeric RNA oligonucleotide mNOX-E36 (aka Spiegelmer) to inhibit MCP-1 has shown promise in murine models of chronic liver disease^22^. Similarly another group has shown that use of the MCP-1 inhibitor 2-Methyl-2-[[1-(phenylmethyl)-1H-indazol-3yl]methoxy]propanoic acid (aka Bindarit) can protect mice when induced with acute pancreatitis, and limit proteinuria in rat models of renal disease^47,48^.

A heightened inflammatory response is common in many LSDs that involve sphingolipid storage as well as other more common diseases^49,50^. In the case of MCP-1 release, one study has showed that C1P can promote MCP-1 release in various cell types via the downstream PI3K/Akt, MEK/ ERK, and p38 pathways^51^. Another study has demonstrated that inhibition of *de novo* synthesized ceramide can lead to IL-6 and MCP-1 secretion from adipocytes *in vitro*^52^. This observation was also replicated in an experiment wherein obese mice, that were treated with myriocin - a potent inhibitor of the *de novo* pathway of ceramide synthesis showed a reduction in ceramide levels and MCP-1 mRNA expression^53^.

Currently the treatment options for FD are limited to anti-inflammatory therapy and, in some patients, BMT. Recombinant acid ceramidase (rhAC) is currently being developed, and has shown great promise as a potential therapy for FD^13^. A recent study showed that treating the ACDase-deficient mice with rhAC significantly improved survival, and reduced MCP-1 with repeated infusions^13^. Still, human studies and clinical approval of this treatment modality are still a ways off. Our study has demonstrated that targeting MCP-1 may delay the course of FD, and contribute to reduced inflammation within the respiratory and visceral systems. While more work will be required, the use of targeted anti-inflammatory treatments in conjunction with substrate reduction therapy, enzyme replacement therapy, or gene therapy may also create a synergistic benefit for FD management^54–57^.

## Material and Methods

### Animal use, breeding and genotyping

To generate homozygous *Asah1*^P361R/P361R^ mice, we crossed *Asah1*^+/P361R^ heterozygotes as previously reported^4^. *CCL2*^tm1Rol^ (MCP-1^−/−^) knockout mice^58^ were purchased from Jackson Laboratory (Bar Harbor, MA). Male MCP-1^−/−^ mice were crossed to female *Asah1*^+/P361R^ mice to generate doubly heterozygous animals (*Asah1*^+/P361R^;MCP-1^+/−^). These doubly heterozygous mice were mated with MCP-1^−/−^ mice to generate *Asah1*^+/P361R^;MCP-1^−/−^ mice. Subsequently *Asah1*^+/P361R^;MCP-1^−/−^ mice were mated with each other to generate *Asah1*^P361R/P361R^ and MCP-1^−/−^ homozygous double mutants (*Asah1*^P361R/P361R^;MCP-1^−/−^). Genotypes were confirmed via PCR using genomic DNA from ear notches. For MCP-1 genotyping, a set of three primers were used, 5-GCC AGA GGC CAC TTG TGT AG-3, 5-TGA CAG TCC CCA GAG TCA CA-3, and 5-TCA TTG GGA TCA TCT TGC TG-3, yielding a 287 bp product for the wild-type gene and a 179 bp product for the knocked-out gene. To detect the wild-type allele of *Asah1*, we used the primers 5-CAG AAG GTA TGC GGC ATC GTC ATA C-3 and 5-AGG GCC ATA CAG AGA AAC CCT GTC TC-3, which yielded a 379 bp product. For the *Asah1* knock-in allele, we used the primers 5-TCA AGG CTT GAC TTT GGG GCA C-3 and 5-GCT GGA CGT AAA CTC CTC TTC AGA CC-3, which amplifies a 469 bp product from the neomycin resistance cassette. All animal procedures were approved and carried out in strict adherence to the policies of the University Health Network (UHN) Animal Care Committee and the Medical College of Wisconsin (MCW) Institutional Animal Care and Use Committee (IACUC).

### Animal data and organ weights

Mice survival was recorded for Kaplan-Meier curves. For organ weight, mice were euthanized with CO_2_ gas and immediately weighed. Organs were collected immediately and weighed. Brain edema was determined by brain wet-to-dry weight ratio. Whole brains were weighed to determine wet weight and then were placed in a laboratory oven (Qualtech Industries Denver, CO) to evaporate for 48 hours. Dried samples were weighed to obtain dry weight. Skin stretch was quantified with a digital vernier caliper (Fisher Scientific, Waltham, MA) by measuring the height of the tent formed when scruffing the mouse.

### Peripheral blood and serum biochemistry, ELISAs, and cytokine analyses

Peripheral blood was collected by cardiac puncture into EDTA-coated microtainers (BD, Biosciences Canada, Mississauga, Canada). Blood samples were inserted into a Hemavet (Drew Scientific Group, Waterbury CT) for complete blood count (CBC) analyses. For serum separation, mouse blood was collected via cardiac puncture into serum separator SST microtainers (BD Biosciences). Sample tubes were gently inverted and the blood was allowed to clot at room temperature for 30 minutes. Samples were then centrifuged at 1200 g for 10 minutes. The serum portion was immediately collected and stored in −80 °C until use. Mouse cytokine levels were measured on collected serum with the Cytokine-20-Plex mouse panel (Thermo Scientific Pierce, Waltham, MA) following the manufacturer’s instructions. Luminescence was measured on the Luminex 100 instrument (Luminex, Austin, TX). Data with a low bead count (<45 beads) was omitted. Additionally, we measured MCP-3 and MCP-5 in mouse serum with MCP-3 and MCP-5 ELISA kits (Thermo Scientific, Waltham, MA). Serum metabolites and liver enzymes in serum were measured using the VetScan Comprehensive Diagnostic Profile and Mammalian Liver Profile (Abaxis Union City, CA) on the Abaxis VetScan VS2 (Abaxis Union City, CA). An AST ELISA kit (Cloud-Clone Corp. Wuhan China) was used to assay for serum amino aspartate transferase (AST) levels.

### BALF turbidity, cytospin and differential

A 20-gauge catheter (BD, Biosciences) was inserted into the trachea of CO_2_-euthanized mice and secured. The lungs were flushed three times by gentle washing with 1.0 ml of ice-cold phosphate buffered saline (PBS). BALF was collected in 1.5 ml tubes and centrifuged at 300× g at 4 °C for 10 minutes. The supernatant was removed and stored at −80 °C. BALF supernatant was measured for turbidity on the NanoDrop One (Thermo Scientific Wilmington DE) at OD_600_. Protein concentration of BALF supernatant was measured using a commercially available bicinchoninic acid (BCA) assay kit (Thermo Scientific Pierce, Waltham, MA). Surfactant proteins A, B, C, and D in BALF supernatant were measured using ELISA kits for mouse material (Cloud-Clone Corp. Wuhan, China) as per the manufacturer’s instructions.

For cell analyses, BALF cell pellets were resuspended in 500 µl of PBS and varying dilutions were used to measure total cell counts, which were performed on trypan blue-stained samples on the Countess II FL automated cell counter (Life Technologies, Carlsbad, CA). The remainder of the resuspended cells were diluted in various concentrations to a total volume of 200 µl with PBS and a cytospin was performed with the Shandon CytoSpin III Cytocentrifuge (Thermo Shandon, Waltham, MA) for 5 mins at 300 g. For cell type analyses, cytospins that contained 200,000 cells per slide were stained with the Kwik-Diff kit (Thermo Scientific Pierce, Waltham, MA). Differential cell counts were obtained by averaging counts from 10 non-overlapping zones with a total of 200 cells scored in each zone.

### Histopathology and immunohistochemistry

As mentioned above, mice were euthanized by CO_2_ inhalation. Cardiac perfusion with ice-cold PBS was performed with a 24 G needle and, following necropsy, organs were immediately fixed in 10% phosphate-buffered formalin for 24–48 hours. Organs were subsequently embedded in paraffin and sectioned at 4 μm. Dissected tibias were decalcified with Cal-Ex II fixative/decalcifier per the manufacturer’s instructions (Fisher Scientific.) prior to embedding and sectioning. For analysis of the hematopoietic system, paraffin-embedded thymus, tibia, and spleen samples were stained with hematoxylin and eosin (H&E). Immunohistochemistry (IHC) was then performed with the following primary antibodies: B220 (BD Biosciences #550286; 1:100), CD3 (Sigma #C7930; 1:1000), and Mac-2 for myeloid cells (Cedarlane #CL8942AP; 1:2000). For liver analyses, tissue sections were stained for H&E and Masson’s trichrome. Liver IHC was performed using the following primary antibodies: anti-mouse neutrophil, clone 7/4 (Cedarlane, Burlington, Canada) and rat anti-mouse Mac-2 (Galectin-3) clone M3/38 (Cedarlane). For brain analyses, tissue sections were stained with H&E and IHC was performed using the following primary antibodies: rabbit anti-ionized, calcium-binding adaptor molecule 1 (Iba-1) (Wako Chemicals USA, Cambridge, MA) and chicken anti-glial fibrillary acidic protein (GFAP) (Aves Lab Inc, Tigard, OR). The following secondary antibodies and kits were then used to detect primary antibodies: rabbit anti-rat IgG, biotinylated (Vector Laboratories, Burlingame, CA); goat anti-rabbit IgG, biotinylated (Vector Laboratories); goat anti-rat IgG, biotinylated (Vector Laboratories); donkey anti-chicken IgG (Jackson ImmunoResearch USA, West Grove PA); donkey anti-rabbit IgG, biotinylated (ImmunoResearch); avidin-biotin/HRP (Vector Laboratories); DAB kit (Vector Laboratories) and Vectastain Elite ABC kit (Vector Laboratories). Histology slides were scanned on the Aperio AT2 histology slide scanner (Leica Biosystems, Buffalo Grove, IL) or NanoZoomer 2.0-HT histology slide scanner (Hamamatsu Photonics, Ichinocho, Japan). Scanned micrographs were analyzed with Aperio ImageScope analysis software (Leica Biosystems, Grove, IL).

### Flow cytometry of hematopoietic cells

Flow cytometry was performed on bone marrow, spleen, and thymus samples. For the latter two, cells were forced through a 40 µm nylon cell strainer in PBS with 2% fetal calf serum (FCS). For the bone marrows, cells were collected by flushing femurs and tibias with PBS containing 2% FCS. After RBC lysis, collected cells were washed and resuspended in PBS with 2% FCS and counted on a hemocytometer. For fluorescence-activated cell sorting (FACS) analyses, cells were stained for 30 mins at 4 °C with the following antibodies: CD3 FITC (BioLegend San Diego, CA; 17A2), CD4 PE (BD Biosciences; GK1.5), CD8 APC (BD Biosciences; 53–6.7), CD19 BV605 (Biolegend; 6D5), CD43 APC (Biolegend; S11), IgD e450 (eBiosciences; 11–26 Waltham, MA), IgM PE-Cy-7 (Biolegend; RMM-1), B220 PE (eBiosciences RA3–6B2), Gr-1 FITC (Biolegend RB-6–8C5), Mac1 PE (BD Biosciences; M1/70), and CD11c APC (eBiosciences; N418). FACS analysis was performed on a LSR Fortessa flow cytometer (BD Biosciences) with FACSDiva software (BD Biosciences). Data was analyzed with FlowJo software (Tree Star Inc, Ashland, OR).

### Sphingolipid mass spectrometry

Brain, liver, and lung tissue samples were homogenized in 500 µl PBS with the Omni Bead Raptor 24 tissue homogenizer (Omni International, Inc., Kennesaw, GA) using 2.8 mm ceramic beads. Lipids were extracted from 50 µl of tissue lysate with 200 µl isopropanol. Two sets of lipid mass spectrometry analyses were performed. In the first experiment; the following internal standards were used for sphingolipid measurements: ceramide 100 ng (d18:1/ 22:0) d_4_ (Medical University of South Carolina (MUSC) Lipidomics Core, Charleston, SC); monohexosylceramide 100 ng (d18:1/17:0) (Avanti Polar Lipids Inc., Alabaster, AL); sphingomyelin, 1000 ng (d18:1/17:0) (Avanti Polar Lipids Inc.); and ceramide-1-phosphate (d18:1/16:0), (d18:1/24:0) and (d18:1/24:1) (Matreya Inc., Pleasant Gap, PA); (Avanti Polar Lipids Inc.)^59^. For the second experiment the following internal standards were used: 100 ng of d_7_-Sphingosine (Avanti Polar Lipids Inc.); and 100 ng of d_7_-sphingosine-1-phosphate (Avanti Polar Lipids Inc.). Relative quantification (i.e. peak:area ratio) was obtained for the analytes in comparison to those from their corresponding internal standards.

Samples were analyzed on the Shimadzu 20AD HPLC system using reverse-phase C18 HPLC columns (Agilent Co., Santa Clara, CA) and a Leap PAL autosampler coupled to a triple quadrupole mass spectrometer (API-4000: Applied Biosystems, Carlsbad, CA) operated in MRM (multiple reaction mode) at the MUSC Lipidomics Core. Positive-ion ESI mode was used to detect all sphingolipids. Tissue extraction samples were injected in duplicate for data averaging. The Analyst 1.5.1 software was used for data analysis (Applied Biosystems). Data are presented as fold change relative to the averaged sphingolipid values detected in samples from 8–9-week-old *Asah1*^+/+^;MCP-1^+/+^ mice.

### Quantification and image analysis

For the quantitative evaluation of GFAP and Iba-1 staining in brain samples, five non-overlapping images (20x magnification, 485 µm × 285 µm) for each anatomical region of interest were obtained from scanned micrographs for each slide. Three consecutive brain sections per mouse were stained, scanned, and analyzed. All quantitation was performed with Fiji-ImageJ analysis software (National Institutes of Health, Bethesda, MD).

### Behavioral tests

Motor coordination and muscle strength were tested with an accelerating rotating rod (rotarod) and grip strength meter. Circadian rhythms were taken into account; all tests were performed between 8 am and 12 pm. To reduce non-study related variability, all tests were performed with the same apparatus in the same room. For the IITC Rotarod (IITC Inc. Life Science, Woodland Hills CA), mice were placed on the device and subjected to acceleration from 4 rpm to 40 rpm for a total of 5 minutes. The time when the mice fell off - measured from start - was then recorded. Animals were subjected to three trials per day for two consecutive days; with the same individual performing all tests. For the grip strength test, each animal was weighed prior to the trial. Both total-limb grip strength and fore-limb grip strength were measured with a digital grip strength meter (Columbus Instruments, Columbus, OH). For this assay, mice were placed on the metal grid and allowed to grip with either all total limbs or just forelimbs. After confirmation of grip, the mouse tail was pulled posteriorly parallel to the grid at a constant rate until release. The grip strength, measured by Gram-force, was recorded for three consecutive trials and then averaged. The grip strength was expressed as mean grip strength relative to weight Gram-force/gram body weight for both total limbs and forelimbs.

### Statistical analyses

Data are expressed as means ± standard error. Survival significance was tested with the log-rank (Mantal-Cox) test comparing pairs of curves. All subsequent data was analyzed with a 1-way ANOVA followed by a Tukey post-test. All statistics were analyzed using GraphPad Prism 5.0 (GraphPad Software Inc, La Jolla, CA). Significant differences are expressed in the figures as *p < 0.05, **p < 0.01, and ***p < 0.001.

## Electronic supplementary material

Supplemental Material PDF
