# Supplementary material for: Deletion of MCP-1 Impedes Pathogenesis of Acid Ceramidase Deficiency
Source: Sci Rep. 2018 Jan 29;8:1808. doi: 10.1038/s41598-018-20052-6 (PMC5789088; doi:10.1038/s41598-018-20052-6)
Supplement: Supplementary file 1 — Supplemental Material PDF [file 41598_2018_20052_MOESM1_ESM.pdf]

Supplementary information

Title: Deletion of MCP-1 Impedes Pathogenesis of Acid Ceramidase Deficiency

Authors: Fabian P.S. Yu[1,4], Shaalee Dworski[1], and Jeffrey A. Medin[1,2,3,4,5]

[1] Institute of Medical Science, University of Toronto, Toronto, ON, Canada,
[2] Department of Medical Biophysics, University of Toronto, Toronto ON, Canada,
[3] University Health Network, Toronto, ON, Canada,
[4] Department of Pediatrics, Medical College of Wisconsin, Milwaukee, WI, USA
[5] Department of Biochemistry, Medical College of Wisconsin, Milwaukee, WI, USA

Corresponding Author: Jeffrey A. Medin, Ph.D.
Email: jmedin@mcw.edu

Supplementary Figure S1. No improvement of disease course in *Asah1*$^{P361R/P361R}$;MCP-1$^{+/-}$ heterozygous mice

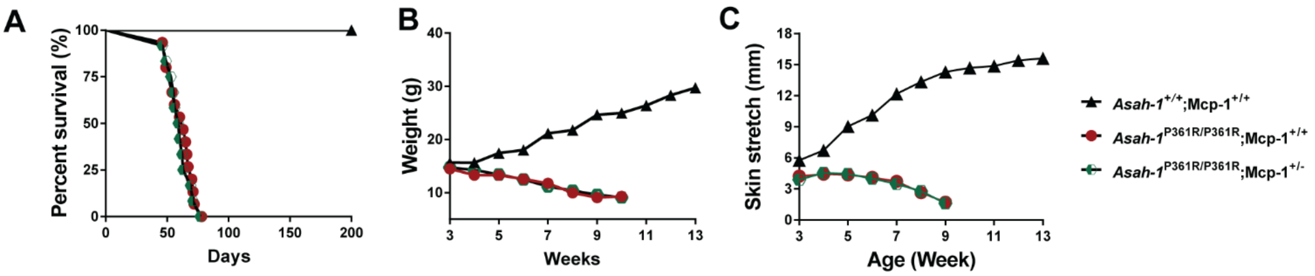

Kaplan-Meier survival plot (*n*=10 per genotype) (A), growth curve measured in weights versus age (*n*=10 per genotype) (B), Skin stretch curves measured as length versus age (n=10 per genotype) (C).

Supplementary Figure S2. Absolute organ weights of mice are largely unchanged in *Asah1*$^{P361R/P361R}$;MCP-1$^{-/-}$ mice

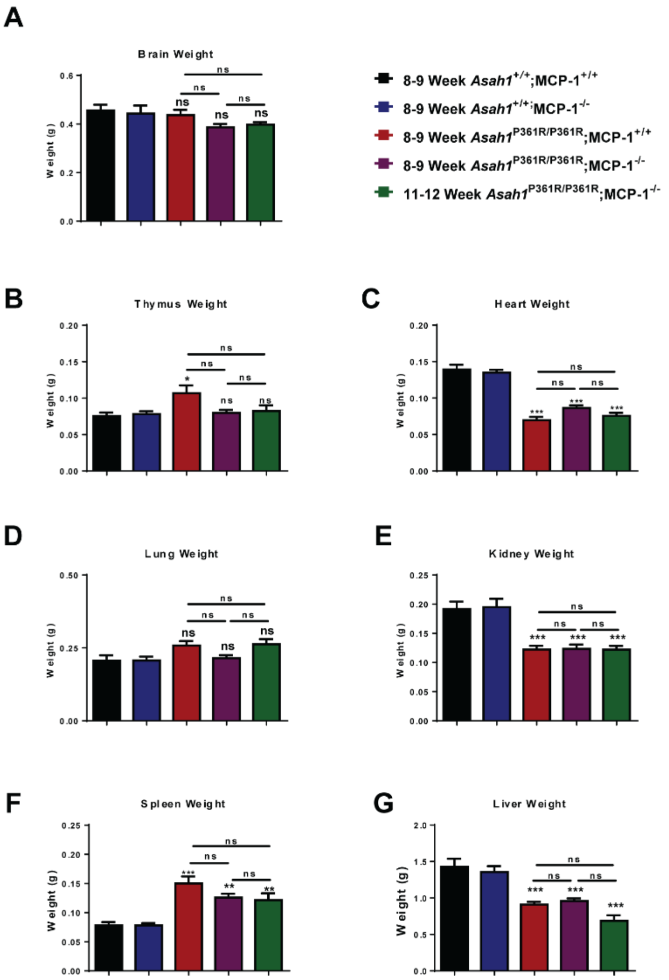

Absolute organ weights for brain (A), thymus (B), heart (C), lung (D), kidney (E), spleen (F), and liver (G). *n*=10 samples were analyzed from 8-9-weeks-old mice for each genotype and from 11-12-week-old *Asah1*$^{P361R/P361R}$;MCP-1$^{-/-}$ mice. All comparisons were made between 8-9-week-old mice for each genotype and samples from 11-12-week-old *Asah1*$^{P361R/P361R}$;MCP-1$^{-/-}$ mice. All comparisons were made between 8-9-week-old *Asah1*$^{+/+}$;MCP-1$^{+/+}$, ns (not significant), **p*<0.05, ***p*<0.01, ****p*<0.001.

Supplementary Figure S3. Absence of T-cell population in *Asah1*$^{P361R/P361R}$;MCP-1$^{-/-}$ mice

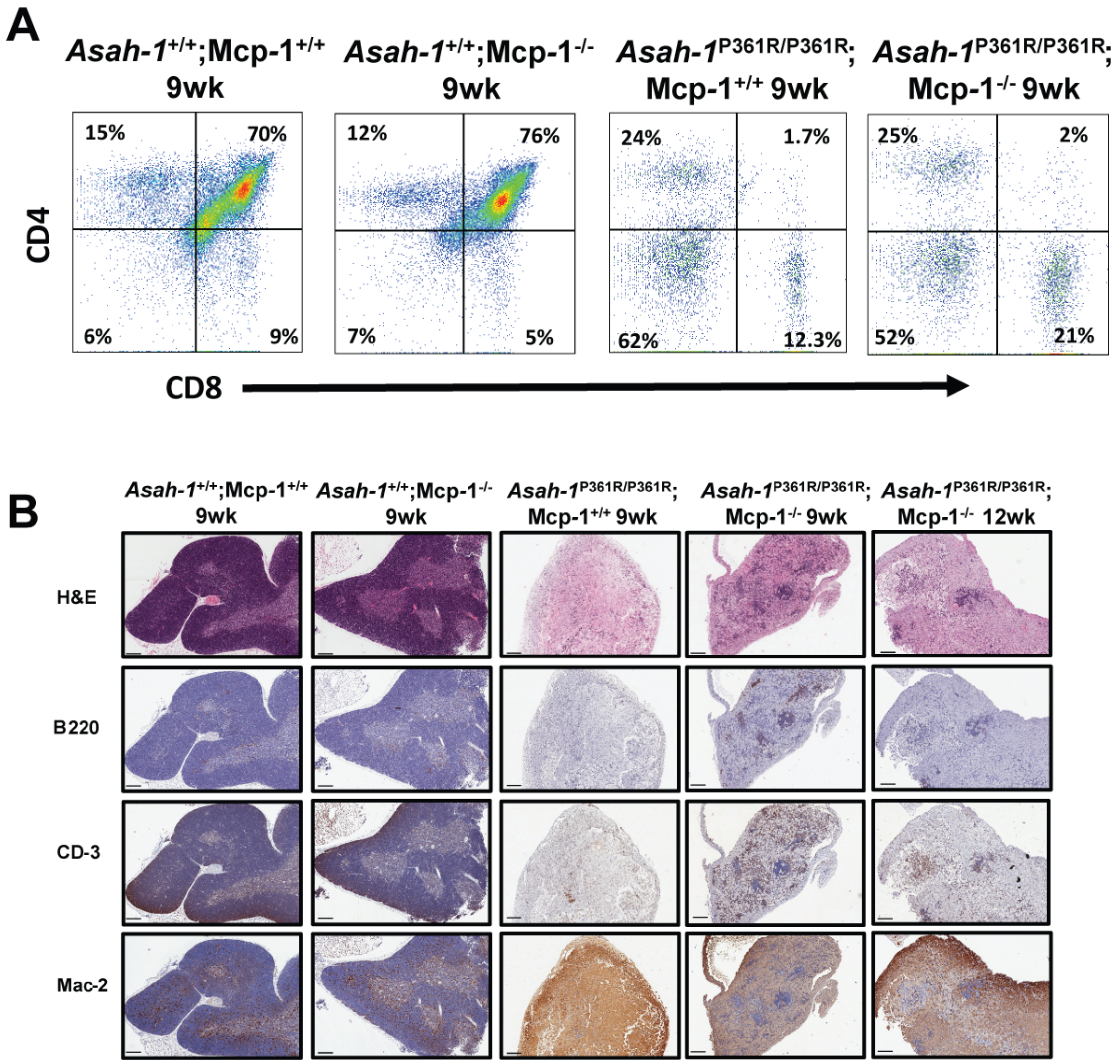

Results of flow cytometry analyses performed on thymus tissue from 9-week-old mice of all genotypes following staining for CD4 and CD8 cells (A). Thymus histology from 9-week-old mice of all genotypes and from 12-week-old *Asah1*$^{P361R/P361R}$;MCP-1$^{-/-}$ mice. Tissues were stained for H&E, B220, CD-3, and Mac-2 (B). *n*=3-4 mice at 9 weeks of age were used for FACS analysis and cell counts.

Supplementary Figure S4. Mild impedance of granulocyte infiltration in *Asah1*$^{P361R/P361R}$;MCP-1$^{-/-}$ mice

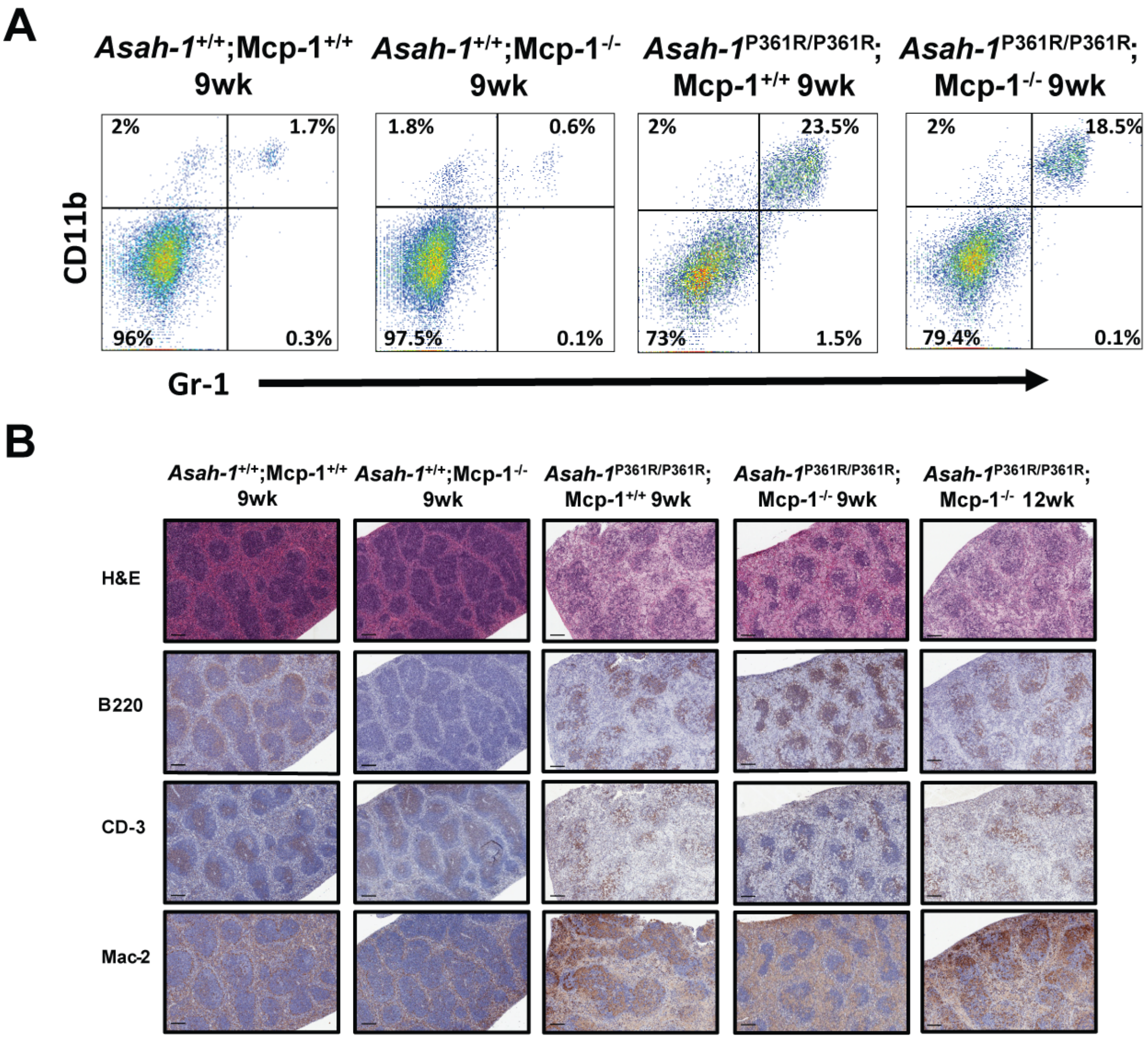

Results of flow cytometry analyses performed on spleen tissue from 9-week-old mice of all genotypes following staining for CD11b and GR-1 cells (A). Spleen histology from 9-week-old mice of all genotypes and from 12-week-old *Asah1*$^{P361R/P361R}$;MCP-1$^{-/-}$ mice. Tissues were stained for H&E, B220, CD-3, and Mac-2 (B). *n*=3-4 mice at 9 weeks of age were used for FACS analysis and cell counts.

Supplementary Figure S5. Monohexosylceramide (MHC) and Ceramide-1-phosphate (C1P) quantification

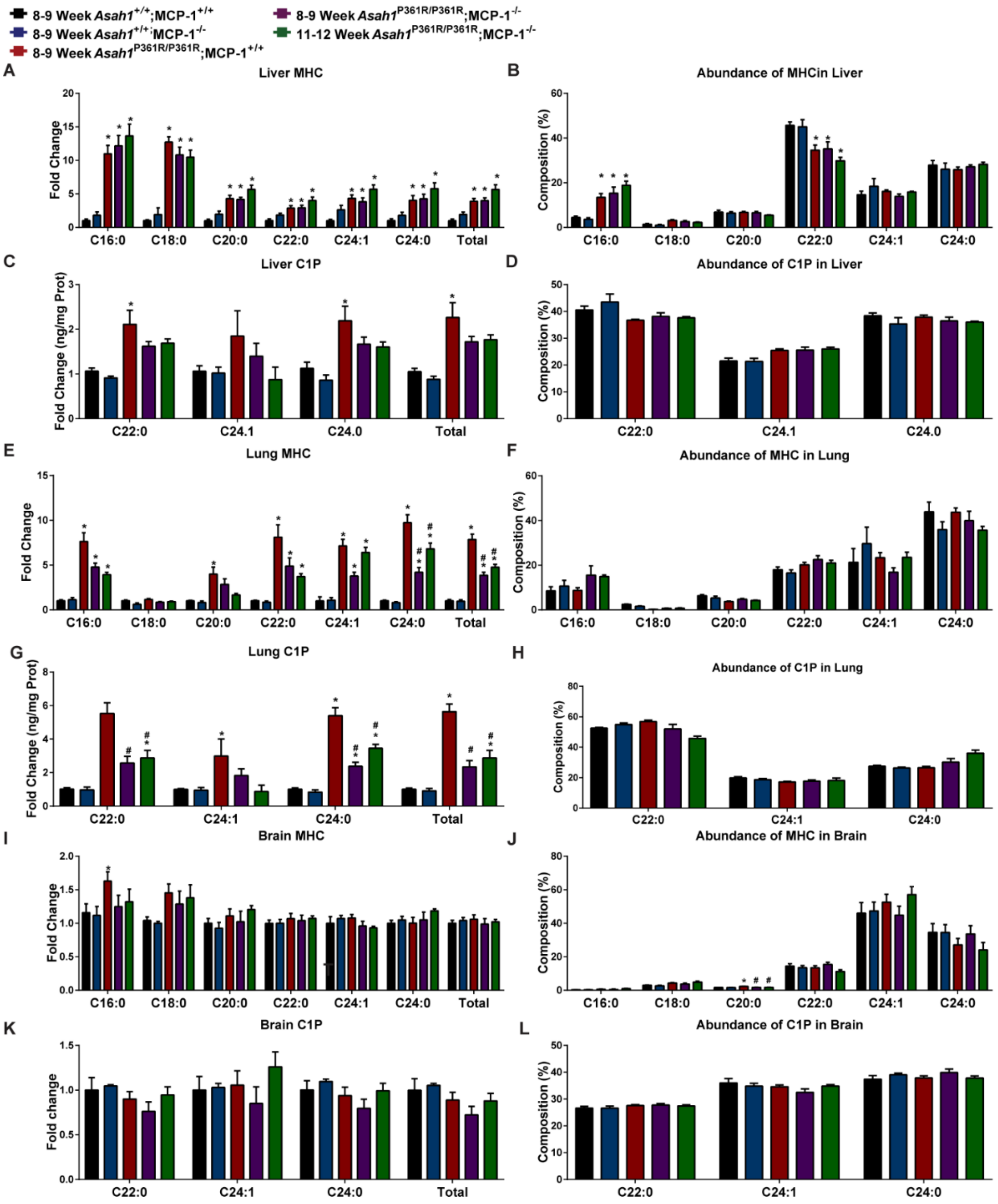

MHC species in liver (A), relative abundance of MHC species in liver (B), MHC species in lung (C), relative abundance of MHC species in lung (D), MHC species in brain (E), relative abundance of MHC species in brain (F), C1P species in liver (G), relative abundance of C1P species in liver (H), C1P species in lung (I), relative abundance of C1P species in lung (J), C1P

species in brain (K), and relative abundance of C1P species in brain (L) were determined. *n*= 4-6 per genotype. All comparisons were made between 8-9-week-old *Asah1*$^{P361R/P361R}$;MCP-1$^{+/+}$, 8-9-week-old *Asah1*$^{P361R/P361R}$;MCP-1$^{-/-}$ and 11-12-week-old *Asah1*$^{P361R/P361R}$;MCP-1$^{-/-}$ mice. ns (not significant), * represent post-hoc test compared *Asah1*$^{+/+}$;MCP-1$^{+/+}$ to *$p<0.05$, **$p<0.01$,***$p<0.001$. $^{\#}$ represent post-hoc test compared to *Asah1*$^{P361R/P361R}$;MCP-1$^{+/+}$ $^{\#}p<0.05$.

Supplementary Figure S6. Sphingosine and Sphingosine-1-phosphate evaluation

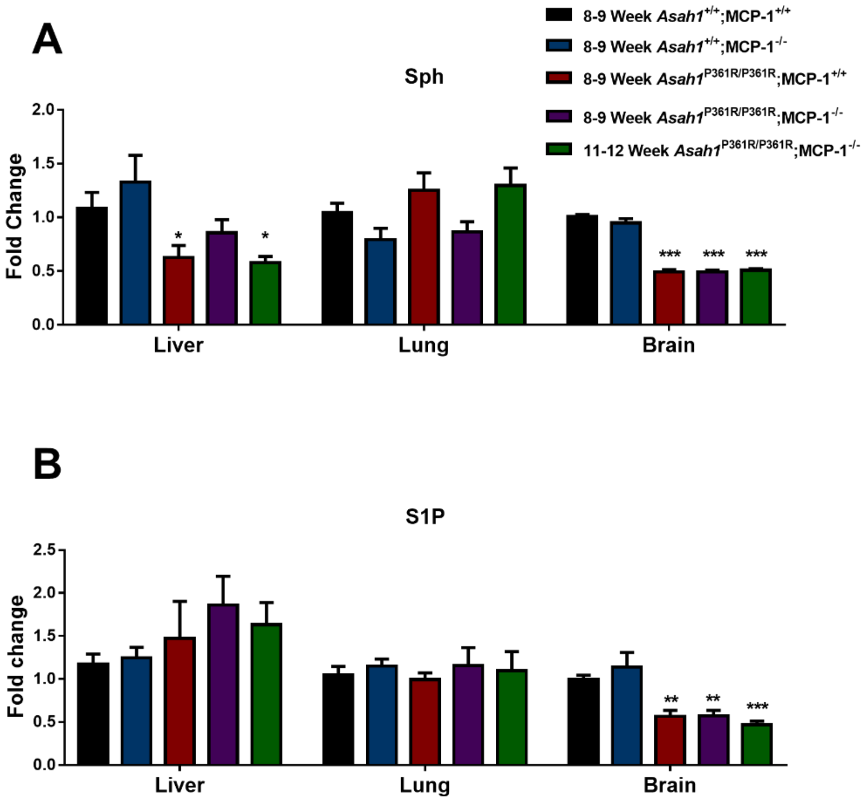

Sph fold change measurements in liver, lung and brain lipid extracts (A). and S1P fold change in liver, lung and brain lipid extracts (B). *n*= 4-6 per genotype. All comparisons were made between 8-9-week-old *Asah1*$^{P361R/P361R}$;MCP-1$^{+/+}$, 8-9-week-old *Asah1*$^{P361R/P361R}$;MCP-1$^{-/-}$ and 11-12-week-old *Asah1*$^{P361R/P361R}$;MCP-1$^{-/-}$ mice. ns (not significant), * represent post-hoc test compared *Asah1*$^{+/+}$;MCP-1$^{+/+}$ to *$p<0.05$, **$p<0.01$,***$p<0.001$.

Supplementary Figure S7. Cytokines that were unchanged in serum analyses.

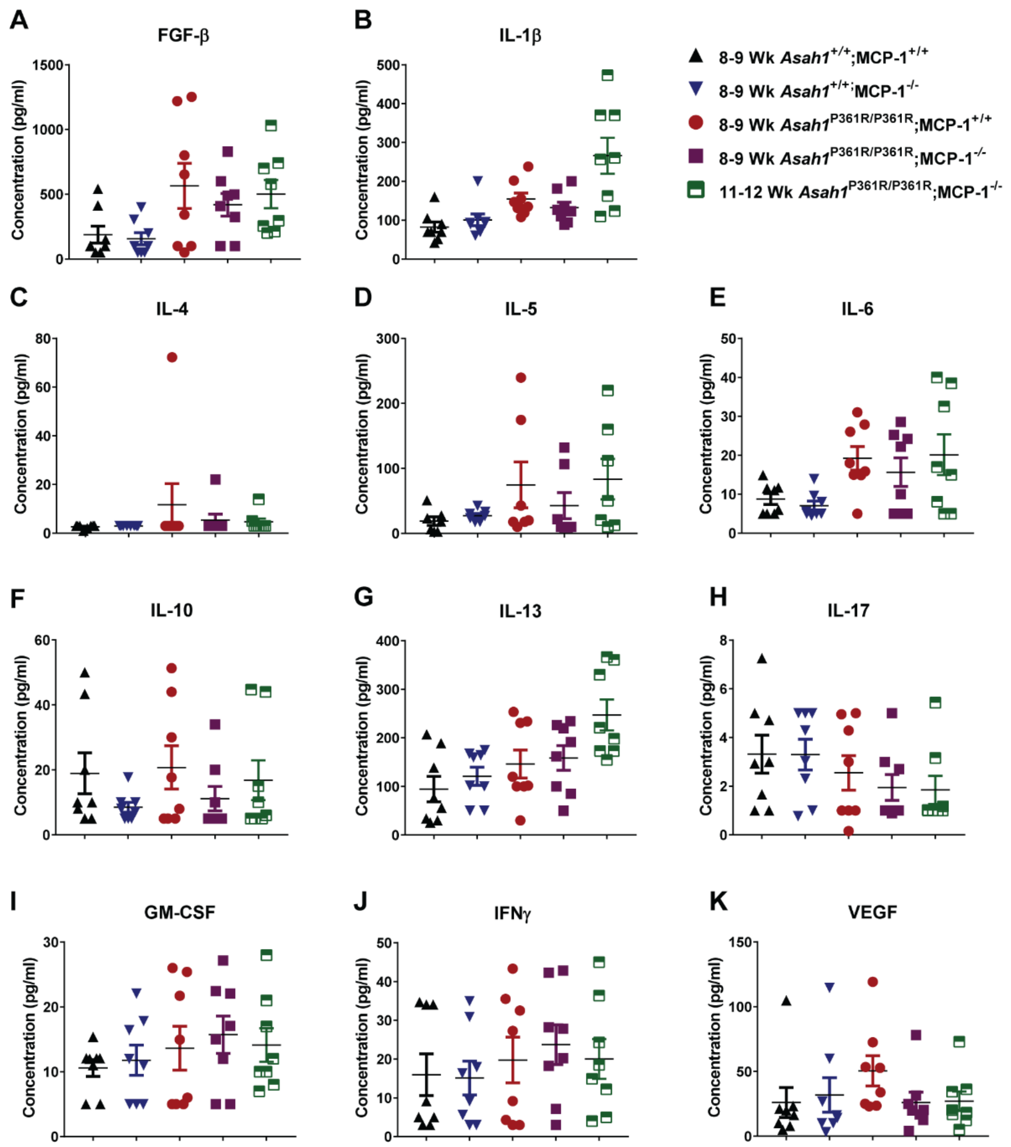

Serum cytokine levels were measured in samples from 8-9-week-old mice for each genotype, and from 11-12-week-old *Asah1*$^{P361R/P361R}$;MCP-1$^{-/-}$ mice. Levels of FGF-β (A), IL-1β (B), IL-4 (C), IL-5 (D), IL-6 (E), IL-10 (F), IL-13 (G), IL-17 (H), GM-CSF (I), IFNγ (J), VEGF (K) were determined. $n = 8$. All comparisons were made between 8-9-week-old *Asah1*$^{+/+}$;MCP-1$^{+/+}$, *Asah1*$^{P361R/P361R}$;MCP-1$^{+/+}$, *Asah1*$^{P361R/P361R}$; MCP-1$^{-/-}$ and 11-12-week-old *Asah1*$^{P361R/P361R}$;MCP-1$^{-/-}$ mice. ns (not significant), *$p<0.05$, ***$p<0.001$.
